# Inverting the structure–property map of truss metamaterials by deep learning

**DOI:** 10.1073/pnas.2111505119

**Published:** 2021-12-30

**Authors:** Jan-Hendrik Bastek, Siddhant Kumar, Bastian Telgen, Raphaël N. Glaesener, Dennis M. Kochmann

**Affiliations:** ^a^Mechanics & Materials Laboratory, Department of Mechanical and Process Engineering, Eidgenössische Technische Hochschule Zürich, 8092 Zürich, Switzerland;; ^b^Department of Materials Science and Engineering, Delft University of Technology, 2628 CD Delft, The Netherlands

**Keywords:** inverse design, truss, metamaterial, deep learning, stiffness

## Abstract

Inspired by crystallography, the periodic assembly of trusses into architected materials has enjoyed popularity for more than a decade and produced countless cellular structures with beneficial mechanical properties. Despite the successful and steady enrichment of the truss design space, the inverse design has remained a challenge: While predicting effective truss properties is now commonplace, efficiently identifying architectures that have homogeneous or spatially varying target properties has remained a roadblock to applications from lightweight structures to biomimetic implants. To overcome this gap, we propose a deep-learning framework, which combines neural networks with enforced physical constraints, to predict truss architectures with fully tailored anisotropic stiffness. Trained on millions of unit cells, it covers an enormous design space of topologically distinct truss lattices and accurately identifies architectures matching previously unseen stiffness responses. We demonstrate the application to patient-specific bone implants matching clinical stiffness data, and we discuss the extension to spatially graded cellular structures with locally optimal properties.

## Significance

More than a decade of research has been devoted to leveraging the rich mechanical playground of periodically assembled truss metamaterials. The enormous design space of manufacturable unit cells, however, has made the inverse design a challenge: How does one efficiently identify a complex truss that has given target properties? We answer this question by a data-driven method, which instantly (once trained, within milliseconds) generates not one but a variety of truss unit cells, whose effective response closely matches a given (fully anisotropic) stiffness tensor. Moreover, our framework to smoothly transition between different unit cells enables the design of lightweight structures with spatially varying, locally optimized properties, for applications from wave guiding to artificial bone.

Opportunities for selecting materials in the engineering design process have fundamentally changed over the past decade due to innovation in materials systems with tailored properties. Specifically with maturing additive manufacturing techniques across length scales, metamaterials or architected materials have gained momentum, whose periodic or nonperiodic structural architecture on smaller scales has enabled us to control the characteristic material behavior on larger scales. Popular examples are periodic assemblies of truss, plate, or shell networks, whose underlying unit cell (UC) architecture can be leveraged to explore a tremendous design space, including previously unattainable effective material property and functionality combinations. This includes lightweight materials with high specific stiffness and strength (1–3), materials for acoustic wave guiding (4–6) and impact energy absorption (7) and further for heat transfer (8–10) and vibration control (11, 12), to mention but a few properties of interest. Beneficial structure–property relations have been identified by user intuition as well as by computational optimization (13) and by taking inspiration from natural cellular architectures (14). While the result is an ever-growing space of candidate UC architectures, the selection process for a specific application too often relies on lookup tables (15) rather than inverse design. The latter is the challenge of identifying architectures that possess specific effective properties on demand (as opposed to the well-understood forward problem of extracting effective properties from a given UC).

To illustrate this challenge and, moreover, to offer a solution, we focus on one of the most fundamental effective properties, the elastic stiffness of a material, which in general is direction dependent or anisotropic. The elastic stiffness not only governs the linear stress–strain response, but also is essential for, e.g., wave motion, buckling, and limit loads. Anisotropic behavior is frequently encountered in nature, e.g., in bone (16). Consequently, material solutions for bone implants should ideally reproduce the mechanical and physiological properties of their natural analogs. This, in turn, requires finding cellular architectures whose effective anisotropic stiffness matches that of bone (which commonly varies not only from patient to patient but even from location to location within a specific bone). If unsuccessful, mismatches in stiffness can lead to stress shielding with detrimental consequences for bone atrophy (17, 18). Prior research has identified a myriad of architectures with tailored anisotropy—behaving stiff in some directions and compliant in others. Most popular for its simple fabrication and property extraction has been the class of truss lattices (13, 18–28). In principle, arbitrary thermodynamically admissible stiffness combinations can be achieved by the complex arrangement of trusses (20), which may be guided by topology optimization schemes (also referred to as inverse homogenization) (13). Most prior work, however, has relied on simple parametric studies (21, 29) (with a limited range of achievable [an]isotropy), structural optimization techniques (13, 27) (which are expensive in three dimensions [3D] and may not guarantee manufacturability), or the selection from a precomputed UC database (14, 15) (which becomes prohibitively large for high-dimensional parameter spaces). Moreover, identified topologies for different elastic stiffnesses are often incompatible and hence not continuously convertible into each other, which prevents their use in structures with spatially varying, locally optimized stiffness, such as in bone.

In recent years, machine-learning (ML) algorithms such as deep neural networks (NNs) have gained attention to meet the inverse design challenge. Existing approaches of single- and multiscale topology optimization commonly leverage data-driven surrogate models for the structure-to-property map, which bypass expensive computational homogenization of the microscale UC and thereby accelerate structural optimization (30–34). Generative models based on variational autoencoders and generative adversarial networks typically search for optimal designs with target properties from within a continuous latent (design) space of reduced dimensionality (23, 28, 35–37). The biggest challenge in inverse-designing truss architectures is the lack of a unifying design parameterization describing the enormous set of truss lattices identified over the years. While a pixelated/voxelated microstructure representation has been successful for, e.g., composite materials (37–40), it is highly inefficient for sparse 3D truss UCs. Other approaches, like the library of tens of thousands of unique truss lattices introduced recently (14) with inspiration from molecular structures, do not admit a consistent design parameterization (unlike their molecular analogs). Consequently, existing works (23, 25, 30, 41) have typically considered only a small number of fixed lattice topologies, whose superposition with different strut thicknesses and/or base materials results in a limited design space for the effective properties—but with the added benefit of enabling spatial gradients. In addition, the common focus on cubic and hence orthotropic UCs (26, 35) ignores shear–normal and shear–shear coupling components in the effective stiffness tensor—although it has been recognized that those may be beneficial for, among others, compliance minimization and wave guidance (22, 42). By contrast, a completely random topology (based on a random placement and connection of struts in a truss) results in an overwhelmingly high-dimensional and nonlinear parameterization with low symmetry and a prohibitively small fraction of mechanically useful UCs (not even to think of smooth spatial transitions between different random topologies). More recently, graph neural networks (leveraging the analogy between trusses and graphs) have shown promising results in predicting the response of truss metamaterials (albeit in a semisupervised setting of the forward problem only), and their use for design optimization is the subject of current research (43).

We here propose an ML-driven inverse design framework for the instant prediction of diverse truss lattices with fully tailorable 3D anisotropic stiffness. Our framework admits an enormous, unified design space of topologically distinct lattices with an efficient design parameterization. The property space spans several orders of magnitude in stiffness, including previously unexploited shear–shear and shear–normal couplings. The inverse design admits variational sampling to propose multiple distinct architectures that exhibit a given target stiffness response—all while maintaining the ability to smoothly transition between different UCs, which is an ongoing challenge for periodic structures (44).

## Creating a Diverse Lattice Design Space

**Fig. 1.**
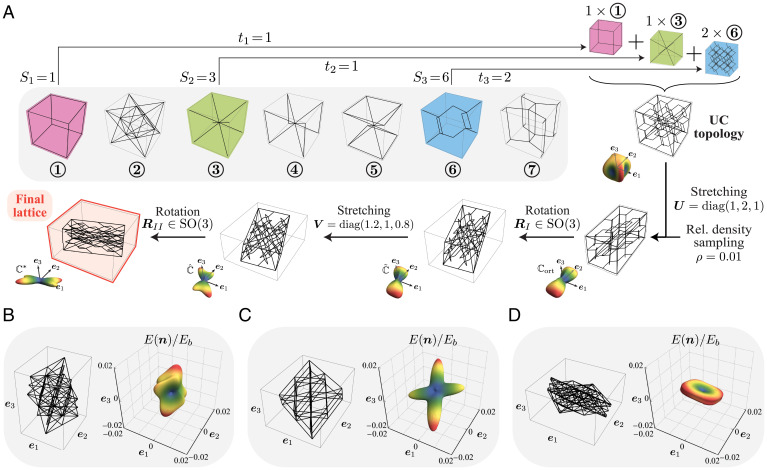
Generation of a diverse truss dataset. (*A*) Sequence of generating different lattice realizations, showing the corresponding elastic surfaces at each stage. First, a topology is randomly drawn by superimposing and tessellating up to three unique elementary topologies (45). Next, its geometry is transformed by four affine deformations to break the orthotropic symmetry and enable shear–shear and shear–normal coupling, while maintaining continuous design/stiffness spaces. Elastic surfaces were obtained by FE simulations and qualitatively illustrate the effective directional Young’s modulus E(n) for all directions $n\in S^{2}$ in the Cartesian basis $\left\{e_{1},e_{2},e_{3}\right\}$. (*B*–*D*) Examples of truss UC realizations, showcasing the diverse elastic responses (normalized by the base material’s Young’s modulus *E_b_*). While all three shown examples have a relative density ρ=0.05, the actual dataset randomly samples *ρ* to cover a large range of stiffness responses.

We start by defining a large, structured design space of truss lattices by drawing inspiration from the truss descriptors proposed by Zok et al. (45). Fig. 1*A* illustrates our approach for generating truss UCs, which differ in their 1) topology, 2) geometry, and 3) relative density. To define the topology, we introduce a set S of seven elementary lattice topologies as fundamental building blocks (details provided in *SI Appendix*, Fig. S1). These comprise both well-studied topologies (like the octet) and nonstandard ones to admit a wide range of anisotropic responses with a relatively small number of beam elements. From this set, we sample three lattices $(S_{1},S_{2},S_{3})\subset S^{3}$ with repetitions allowed, each of which is placed inside a unit cube either as a 1×1×1 tessellation or as a 2×2×2 tessellation (for short, denoted by 1× or 2×, respectively). Superimposing the three lattices yields a compound lattice $t_{1}\times S_{1}+t_{2}\times S_{2}+t_{3}\times S_{3}$ with $t_{i}\in \{1,2\}$. The topological design parameters $\left(S_{1},S_{2},S_{3},t_{1},t_{2},t_{3}\right)$ result in a set of 262 unique topologies.* Any intersections of struts during the superposition of the elementary lattices are resolved by splitting the affected struts and introducing a new vertex at the intersection.

Owing to the symmetries of the seven chosen elementary lattices, all resulting lattices are orthotropic (i.e., their 3D stiffness tensors have only nine independent constants). To expand the achievable stiffness space in a continuous manner, we enlarge the design space by applying a series of affine geometric transformations to the obtained lattice topology, which transforms every vertex at a location $X_{j}$ to its new location $x_{j}$ as follows: Assuming that the UC aligns with the Cartesian coordinate axes $\left\{e_{1},e_{2},e_{3}\right\}$, we first stretch the UC with a stretch tensor $U=\text{diag}(U_{1},U_{2},U_{3})$ with principal stretches $U_{1},U_{2},U_{3}>0$, followed by a rigid-body rotation $R_{I}\in \text{SO}(3)$. Next, we apply another round of stretches via $V=\text{diag}(V_{1},V_{2},V_{3})$ with eigenvalues $V_{1},V_{2},V_{3}>0$ and a rigid-body rotation $R_{II}\in \text{SO}(3)$, both with respect to the same (original) reference frame $\left\{e_{1},e_{2},e_{3}\right\}$. This four-stage process affinely transforms the original UC, such that any vertex position X is mapped to

[1] $$x=R_{II}VR_{I}\mathrm{UX}.$$

Note that U stretches the lattice along its orthotropic symmetry planes, while the combination of $R_{I}$ and V shears the lattice, thus breaking the orthotropic symmetry and introducing shear–shear and shear–normal coupling (characterized by 21 independent constants in the 3D stiffness matrix). The final rotation $R_{II}$ allows for orienting the UC arbitrarily in 3D space.

A rich set of anisotropic lattices is created by generating random transformations for each of the 262 topologies introduced above. To obtain physically reasonable lattices, all principal stretches are sampled from the range $U_{i},V_{i}\in [0.5,2]$. These six stretches, together with the six parameters describing rotations $R_{I}$ and $R_{II}$ (each characterized uniquely by three parameters in the axis-angle representation in 3D), thus constitute the geometrical design parameters, which can be applied to any given topology. As shown in Fig. 1*A*, this drastically influences the 3D stiffness response and lets us access an expanded domain in the stiffness space.

Finally, we vary the relative density (i.e., the fill fraction of the UC) in the range ρ∈[0.002,0.1] by controlling the uniform thickness *d* of all struts in the UC (assuming circular cross-sections). Jointly with Young’s modulus *E_b_* of the base material (which we assume to be isotropic with Poisson’s ratio ν=0.3), *ρ* essentially affects the effective UC stiffness—not only its magnitude but also its anisotropy by controlling the balance between bending—vs. stretching-dominated deformation.^†^ As the latter depends on the UC topology, there unfortunately is no simple scaling law to predict the effective UC response (14, 46), which necessitates mechanical homogenization simulations.

A major advantage of the above explicit truss descriptor becomes obvious when computing the homogenized response. Unlike voxel-based approaches, whose required resolution quickly becomes computationally infeasible in 3D (35), the truss-based description allows for efficient computations, in which each strut is modeled as a linear elastic Timoshenko beam (which is sufficient for the considered small-strain response). We use a finite-element (FE) setting with periodic boundary conditions applied to the UC (*SI Appendix*, section 2) to extract the homogenized stiffness tensor (47), producing a dataset of millions of randomly generated lattices along with their corresponding (physically admissible) homogenized stiffness components ${\mathbb{C}}_{\mathrm{ijkl}}$ (i,j,k,l∈{1,2,3}), satisfying positive definiteness, having symmetries ${\mathbb{C}}_{\mathrm{ijkl}}={\mathbb{C}}_{\mathrm{jikl}}={\mathbb{C}}_{\mathrm{klij}}={\mathbb{C}}_{\mathrm{klji}}$ (48), and honoring the Voigt upper bound for the maximally achievable stiffness for a given relative density. Owing to the linear scaling, we normalize all stiffness values by *E_b_*.

We emphasize that our design and stiffness spaces of truss lattices (categorized into 262 unique topologies) are vastly larger than those of the closest comparable approaches (19, 21, 49), which examined a small set of topologies and limited the property space by varying only the relative density of elementary building blocks. As illustrated by the examples in Fig. 1 *B*–*D*, our design space includes a wide range of anisotropic (nonorthotropic) responses by combining topological and geometrical manipulations of the UC. Of course, our design parameterization is not unique (there is flexibility in the selection of elementary lattices and the parameterization of the affine transformations); the main idea is to offer an elegant and, most importantly, sufficiently rich design space that covers a sufficiently large anisotropic stiffness space.

## An Inverse Design Framework with Physics-Guided Stochastic Deep Learning

While FE homogenization provides an accurate forward model that maps design parameters onto stiffness responses, the inverse problem has remained a challenge. We therefore introduce an inverse design framework that can efficiently reverse engineer a UC design, whose stiffness approximates any (physically admissible) 3D anisotropic stiffness tensor characterized by its 21 independent elastic constants. To train the inverse framework, we create a large dataset of truss lattices, $D=\left\{\{\Theta_{i},{\mathbb{C}}_{i}^{*}\},i=1,\ldots ,n\right\}$, consisting of n=3,000,000 pairs of 1) design parameters Θ (details provided in *SI Appendix*, section 1) and 2) corresponding true stiffness ${\mathbb{C}}^{*}$.

The inverse design problem is inherently ill-posed, as multiple combinations of design parameters may represent the same UC and, moreover, multiple distinct UCs may produce the same effective stiffness. For example, due to the cubic symmetry group of our elementary cells, all rotations $R_{I}$ about a coordinate axis $e_{i}$ with angle kπ, k∈ℤ correspond to the same UC and stiffness response. Apart from the inherent degeneracy of our lattice descriptor, physically different lattices may show similar anisotropic stiffness responses. Hence, going the direct route of predicting a lattice described by Θ as a function of the target anisotropic stiffness ℂ by minimizing the prediction loss of an NN G:ℂ→Θ parameterized by weights and biases ω on the dataset D as

[2] $$G\leftarrow \underset{\omega }{\min}\frac{1}{n}\sum_{i=1}^{n}{\left\|G_{\omega }[{\mathbb{C}}_{i}^{*}]-\Theta_{i}\right\|}^{\;2}$$

is ill-posed (28) due to the one-to-many mapping from ℂ to Θ. Consequently, the NN incorrectly penalizes potentially correct designs and leads to a poorly trained inverse model, irrespective of the complexity of the NN.

**Fig. 2.**
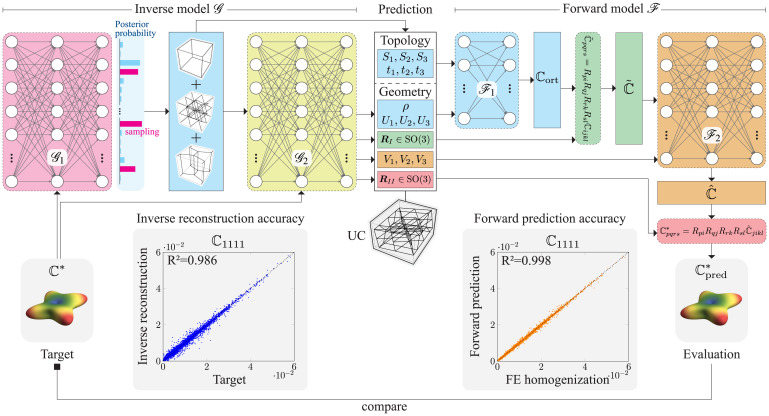
Framework to generate truss lattices with given stiffnesses. The inverse model takes the 21 independent elastic constants of ${\mathbb{C}}^{*}$ as input and first predicts a posterior distribution of possible lattice topologies, from which one (composite) topology is sampled and passed, jointly with ${\mathbb{C}}^{*}$, to a second NN, which predicts the corresponding geometrical parameters. The stiffness of the proposed lattice candidate is then reconstructed with the (independently trained) forward model and compared to the target stiffness. Predictions for ${\mathbb{C}}_{1111}$ of the forward and inverse models are compared to the actual stiffness (obtained via FE modeling and the forward model, respectively) and the corresponding *R*^2^ deviations indicated (evaluated on a test set of 30,000 truss lattices). Further details of the NN architecture, training schemes, and accuracy are summarized in *SI Appendix*, section 3.

To bypass this challenge, we propose an inverse design framework based on a combination of stochastic and physics-guided NN models, which takes inspiration from dual-network models (28, 50, 51) so far used only outside the context of trusses. Fig. 2 illustrates the ML architecture. Instead of training for an accurate prediction of design parameters Θ as in Eq. **2**, we aim for an accurate reconstruction of the stiffness response of the predicted lattice with respect to a given target stiffness. This defines our inverse model and is in line with our original goal of identifying a truss architecture whose response matches a given stiffness—not restricting the prediction solely to the same truss that was used to generate said stiffness in the precomputed dataset.

To efficiently train the inverse model G:ℂ→Θ (mapping from a given stiffness to a potential lattice candidate), we introduce a separate forward model F:Θ→ℂ (mapping from a given lattice to its stiffness). The latter is an efficient surrogate for FE homogenization and reconstructs the stiffness of the truss proposed by the former, thus enabling a direct comparison with the target stiffness. In addition, the forward model provides the ability to track gradients via automatic differentiation, which is crucial for the backpropagation algorithm (52) used to train the inverse model (28). The forward model F, parameterized by weights and biases τ, is trained to minimize the prediction error in the stiffness as

[3] $$F\leftarrow \underset{\tau }{\min}\frac{1}{n}\sum_{i=1}^{n}{\left\|F_{\tau }\left[\Theta_{i}\right]-{\mathbb{C}}_{i}^{*}\right\|}^{\;2}.$$

Once the forward model has been trained, the inverse model is subsequently trained to minimize the reconstruction error (computed by F) in the stiffness of the predicted UC (computed by G) as

[4] $$G\leftarrow \underset{\omega }{\min}\frac{1}{n}\sum_{i=1}^{n}{\left\|F\left[G_{\omega }\left[{\mathbb{C}}_{i}^{*}\right]\right]-{\mathbb{C}}_{i}^{*}\right\|}^{\;2}.$$

Note that the pretrained forward model is not updated during training of the inverse model.

Unlike Eq. **2**, Eq. **4** provides a coherent measure of loss, which penalizes only deviations from the target stiffness. Using a standard “black box” approach of directly passing the design features or stiffness labels into an NN architecture, however, did not yield sufficiently accurate results for both forward and inverse models despite a wide range of network hyperparameters (such as the depth and width of the underlying multilayer perceptrons) being optimized for. Instead, we observed major improvements in accuracy by introducing physically interpretable NNs as subnetworks within both forward and inverse models, as illustrated in Fig. 2. These mimic the proposed four-stage UC descriptor (Fig. 1*A*), as we explain in the following.

### The Forward Model

The forward model F is a composition of two subnetworks. We first trained a subnetwork $F_{1}$ to predict an intermediate stiffness ${\mathbb{C}}_{\text{ort}}$ solely based on the UC topology [described by a selection of the three elementary lattices $\left(S_{1},S_{2},S_{3}\right)$ and their tessellations $\left(t_{1},t_{2},t_{3}\right)$, which are represented as one-hot encoded binary vectors], as well as the relative density *ρ* and eigenvalues ($U_{1},U_{2},U_{3}$) of the first stretch tensor—without introducing any rotation or shear. Therefore, ${\mathbb{C}}_{\text{ort}}$ is orthotropic and represented compactly by only nine nonzero stiffness constants. Subsequently, we transform ${\mathbb{C}}_{\text{ort}}$ using the rotation $R_{I}$. [Fourth-order tensor ℂ transforms under R∈SO(3) as ${\tilde{\mathbb{C}}}_{\mathrm{pqrs}}=R_{pi}R_{qj}R_{rk}R_{sl}{\mathbb{C}}_{\mathrm{ijkl}}$ using Einstein’s summation convention; in practice, the rotational transformation is implemented using Voigt notation (53) for computational efficiency (*SI Appendix*, section 3.B)]. The resulting stiffness tensor $\tilde{\mathbb{C}}$ is passed as an input feature—together with the given eigenvalues $\left(V_{1},V_{2},V_{3}\right)$ of the second stretch tensor—into a second subnetwork $F_{2}$, which is solely trained on the task of identifying the sheared stiffness tensor $\hat{\mathbb{C}}$ and does not need to learn the intricate rotational transformations of higher-order tensors. As the last step, we compute the final rotation of the sheared stiffness tensor $\hat{\mathbb{C}}$ by $R_{II}$ (analogous to $R_{I}$). Most importantly, we continue tracking the computational graph of all these operations (including rotational transformations of intermediate stiffness tensors) to allow for automatic differentiation, which is necessary for the gradient evaluation in the training stage of the inverse model. Subnetworks $F_{1}$ and $F_{2}$ within the forward model are trained independently, each with the mean-squared error as loss function between predicted and ground-truth stiffness (consisting of the 9 orthogonal and full 21 elastic constants in the first and second subnetworks, respectively). As shown in the example correlation plot between the true and predicted stiffness responses of an unseen test set for ${\mathbb{C}}_{1111}$ in Fig. 2 (from the forward model), this setup is sufficiently accurate to evaluate the stiffness response of the predicted lattices, verified by the coefficient of determination $R^{2}\geq 0.98$ across all elastic constants (see *SI Appendix*, Fig. S3 for details). We highlight that, unlike the presented architecture, a single neural network—without any embedded information about the lattice generation process—fails to learn the structure-to-property map. Leveraging the existing knowledge underlying the provided data to enable or accelerate the deep-learning framework aligns with the spirit of recent applications of deep learning to physical problems (54, 55).

### The Inverse Model

Like the forward model, the inverse model G includes two subnetworks—the first subnetwork $G_{1}$ predicts a topology, which is later geometrically manipulated by the second subnetwork $G_{2}$ to closely match the given target stiffness. $G_{1}$ takes the target stiffness as input and predicts the choice of the elementary lattices ($S_{1},S_{2},S_{3}$) and their tessellations ($t_{1},t_{2},t_{3}$), which jointly define the topology of the UC. Since these are categorical design parameters, we expect discrete (one-hot) encoded outputs. We hence pass the predictions of the penultimate NN layer through a softmax layer (56) with a fixed temperature *τ*, the output of which is interpreted as a probability distribution. For a categorical target output with *k* classes, the softmax outputs the probability of each class as

[5] $$p_{i}=\frac{\exp\;(-q_{i}/\tau )}{\sum_{j=1}^{k}\exp\;(-q_{j}/\tau )}\;\text{for}\;i=1,\ldots ,k,$$

where $\left\{q_{i}:i=1,\ldots ,k\right\}$ are the outputs from the penultimate layer of $G_{1}$. This classical setup deterministically collapses to predicting the categorical output with the highest posterior probability—however, we suspect that multiple topologies (with a corresponding set of geometrical parameters) may be equally suited to match the given stiffness and thus share similar posterior probabilities. To access these predictions, we allow for stochastic sampling via the so-called Gumbel-Softmax trick (57). Given the probabilities $\left\{p_{i}:i=1,\ldots ,k\right\}$ from the softmax layer, we draw a sample *z* as

[6] $$z=\arg\;\underset{i}{\max}(g_{i}+\log\;p_{i}),$$

where $\left\{g_{i}:i=1,\ldots ,k\right\}$ are independent and identically distributed samples drawn from the Gumbel distribution via $g_{i}=-\log\;(-\log\;(x_{i}))$ with uniformly distributed $x_{i}∼U[0,1]$. While we use the arg max operator in the forward pass to constrain the sampling to physical (i.e., discrete) predictions only, we allow for differentiation via backpropagation in the backward pass by replacing it with

[7] $$z_{i}=\frac{\exp\;\left[(g_{i}+\log\;p_{i})/\tau \right]}{\sum_{j=1}^{k}\exp\;\left[(g_{j}+\log\;p_{j})/\tau \right]}\;\text{for}\;i=1,\ldots ,k,$$

also known as the straight-through Gumbel estimator (57). This allows for the straightforward use of the reparameterization trick (50) typically found in variational autoencoders, which permits backpropagation through stochastic nodes by differentiating only with respect to the parameters of the distribution but not the sampling (i.e., the Gumbel noise) itself. For τ→0, we recover the categorical distribution *p_k_*, while for larger *τ* we approach a uniform distribution over all categories. We fixed *τ* = 1 during our training process.

The sampled topology (from the Gumbel Softmax) and the target stiffness are jointly passed to the second subnetwork $G_{2}$, which learns the geometric manipulations required to achieve the target stiffness. Outputs of $G_{2}$ are the relative density *ρ*, the six eigenvalues of the stretch tensors U and V, and the rotation matrices $R_{I}$ and $R_{II}$. While any rotation SO(3) is uniquely determined by three independent constants, any such parameterization is discontinuous and challenging for NNs to learn. We therefore use a continuous six-dimensional representation (58) per rotation to efficiently learn rotation matrices using NNs (*SI Appendix*, section 3.B). Note that, contrary to the forward model, both subnetworks $G_{1}$ and $G_{2}$ of the inverse model must be trained simultaneously, as we evaluate only the stiffness of the fully assembled UC, which includes topological and geometrical design parameters.

The reconstruction accuracy of the inverse model for, e.g., ${\mathbb{C}}_{1111}$ is included in Fig. 2, where we show the correlation plot between the target (${\mathbb{C}}^{*}$) and reconstructed stiffness (of the UC with $\Theta =G[{\mathbb{C}}^{*}]$), evaluated on the same unseen test set used for the forward model, but not considering the given design parameters. The inverse model accurately identifies lattice candidates with constitutive properties closely matching the target stiffness, demonstrated by $R^{2}\geq 0.95$ across all stiffness components (the dominant components ${\mathbb{C}}_{1111},\;{\mathbb{C}}_{2222}$, and ${\mathbb{C}}_{3333}$ have a higher $R^{2}\approx 0.985$ compared to the less influential shear–shear couplings with a lower $R^{2}\approx 0.95$; *SI Appendix*, Fig. S4).

Our variational inverse model (enabled by the Gumbel Softmax) attempts to find the underlying categorical probability distribution for candidate topologies matching a target stiffness and may propose different design realizations, giving the user freedom in choosing an appropriate UC, considering, e.g., manufacturability or secondary functions of importance beyond the target stiffness. To give a concrete example, experimental studies have shown that the distances between struts (which naturally vary with topology) are commensurate with the pore size of bone, which plays a key role in transporting nutrients into bone (and bone implants) for cell growth and also for bone ingrowth (59). Our results (presented in *SI Appendix*, section 3.E) show that, on average, only one in 100 of the inverse-designed truss lattices share the same topology as the lattice from the training set, from which the target stiffness was computed. Notably, repeated drawing of inverse predictions for the same test set with a different random seed gives, on average, in approximately two out of three draws a different lattice realization (setting *τ* = 1), which highlights the generative nature of the inverse variational model. Additionally, this corroborates that Eq. **2** is ill-posed and restrictive.

## Generalization to outside the Training Domain, Artificial Bones, and Spatial Grading

**Fig. 3.**
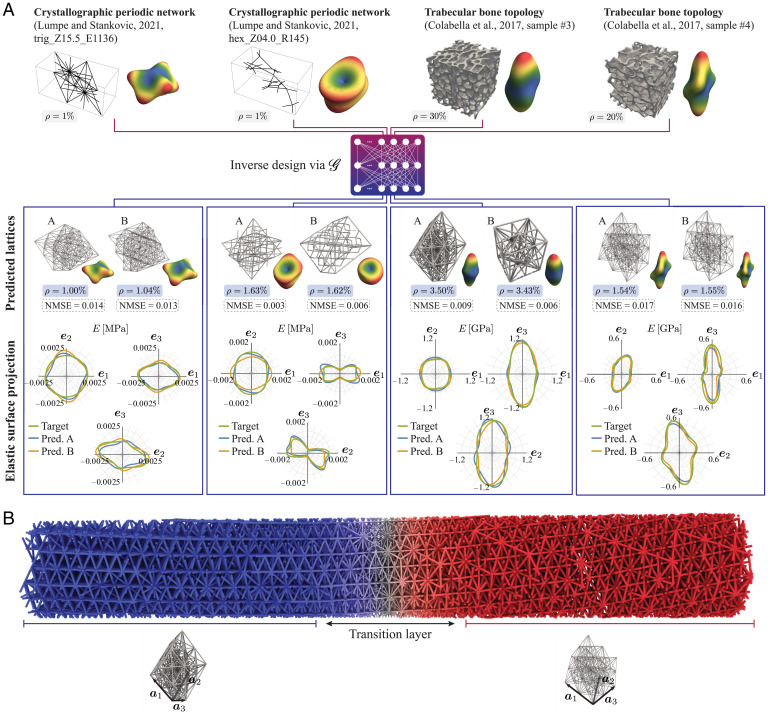
Inverse-designed truss metamaterials: generalization beyond the training dataset and functional grading. (*A*) Inverse design with queries from outside the generated dataset and the corresponding predicted lattice candidates including their elastic surfaces. Two examples are chosen from each of the crystallography-inspired catalogs of periodic truss architectures of ref. 14 and the bovine femoral bone specimens of ref. 16, whose 3D anisotropic stiffness is used as a target for the inverse design. The performance of the inverse design framework is illustrated by projections of the elastic surfaces of the target stiffness and the inverse prediction onto the $e_{1}$-$e_{2},\;e_{1}$-$e_{3}$, and $e_{2}$-$e_{3}$ planes. Two unique designs (denoted by A and B) are predicted (Pred.) for each target to showcase the variational sampling of the inverse design framework. Microtomography images are adapted with permission from Springer Nature: ref. 16. (*B*) A functionally graded architecture is generated by interpolating between the two predictions for the queried bone samples, locally closely matching the given anisotropic stiffness (details on the predictions and interpolation scheme are given in *SI Appendix*, sections 4 and 5). Struts are shown twice as thick for improved visibility.

To assess the performance of our inverse model on stiffness queries outside the proposed lattice design space, we consider two test scenarios. First, we evaluate its performance on randomly drawn (normalized) stiffnesses from a recently reported catalog (lacking a design parameterization) of over 17,000 unique topologies and corresponding stiffnesses based on crystallographic network topologies (14), a few representative results of which are presented in Fig. 3*A* (further evaluations on the full dataset are presented in *SI Appendix*, section 4). We also provide the normalized mean-squared error (NMSE), defined as

[8] $$\text{NMSE}({\mathbb{C}}^{\text{target}},{\mathbb{C}}^{\text{pred}.})=\sum_{i,j=1}^{6}\frac{{\left({\mathbb{C}}_{ij}^{\text{target}}-{\mathbb{C}}_{ij}^{\text{pred}.}\right)}^{2}}{{\left({\mathbb{C}}_{ij}^{\text{target}}\right)}^{2}},$$

where ${\mathbb{C}}_{ij}^{\text{target}}$ and ${\mathbb{C}}_{ij}^{\text{pred}.}$ are, respectively, the stiffness components of the target and inversely predicted designs (using Voigt notation). This allows for a quantitative comparison as well as a further improvement of our inverse predictions: Owing to the generative model of our inverse framework, we may obtain different lattices for different random seeds. Once the networks are trained (*SI Appendix*, section 3.H), we can hence efficiently sample a large variety of lattice candidates and select the one(s) with the lowest NMSE (*SI Appendix*, section 4). The resulting inverse model successfully proposes different UCs of distinct topologies, which closely match the target anisotropic stiffness. We note that, if more exotic anisotropic stiffnesses cannot be matched within our design space, one may add elementary lattices to S to further enrich the design space. (While the training of the NNs may become more intricate due to the higher dimensionality of our design space, we do not foresee fundamental limitations in increasing the design space in this manner.) Besides, we compared the obtained predictions to the best match of the training set to assess the performance of our inverse model as opposed to a lookup-table approach. Results show that the inverse model can identify lattices much closer to a given anisotropic response than the closest match in our given database (*SI Appendix*, section 3.G).

Second, we apply our design framework to synthetic bone. The anisotropic stiffness of an anatomical site on a bone sample provides the gold standard for an ideal bone implant, which avoids stress shielding and improves long-term compatibility (17, 18). To test our inverse design, we take experimentally measured anisotropic stiffness data of trabecular bone (16) as the target. We consider all 21 elastic constants (not restricting to orthotropic symmetry, as done before) (16, 28) and Ti-6Al-4V with Young’s modulus E=114 GPa (60) as base material, which is used for implants due to its excellent biocompatibility and corrosion resistance (17). The results in Fig. 3*A* confirm that the inverse model generates UCs whose effective stiffness closely matches the target 3D anisotropic response (details on the inverse predictions can be found in *SI Appendix*, section 4 and Tables S9–S12). It is hard to give a general guarantee that the network can find designs for any arbitrary (physically admissible) choice of the 21 elastic constants. Yet, the framework performs very well for all reasonable combinations tested (*SI Appendix*, section 4). Furthermore, we can always obtain an estimate of the performance of the generated designs by using the forward model, which warns us if the generated structure is not able to mimic the given stiffness (e.g., if it is physically inadmissible).

Compared to competing methods, the proposed inverse model provides several advantages. First, the data-driven framework is highly efficient and predicts truss lattices corresponding to a prescribed 3D stiffness tensor in fractions of a second (e.g., evaluating a test set of 30,000 stiffness inputs in less than 1 min). This is orders of magnitudes faster than, e.g., classical topology optimization techniques, which iteratively optimize within an extremely large design space (restarting for every given target stiffness), typically by discretizing the UC domain into large numbers of voxels (61). Compared to more recent methods, it also avoids the need for iterative optimization in a latent space required to identify a structure with the target properties (35). Another advantage of the proposed truss-based descriptor lies in the guaranteed manufacturability, as all predicted UCs are composed of simple structural elements and hence achievable by additive manufacturing, which is not guaranteed in general for voxel-/pixel-based architectures (35, 37). Furthermore, such space-filling architectures typically have lower bounds on the relative density (since bicontinuity cannot be ensured and disjoint solid domains may result below a certain density) (28, 62), which makes those architectures infeasible for lightweight structures made of stiff base materials such as Ti-6Al-4V for surgical implants. We emphasize that every inverse prediction corresponds to a physical lattice, since our categorical variables can take only discrete outputs. This bypasses postprocessing steps necessary in approaches that optimize within a latent design space, where not every point may correspond to a physical configuration (23).

A further advantage of the chosen design space lies in its applicability to spatially graded trusses. By introducing a smooth functional grading between any set of predicted UCs, we enable the generation of larger structures with locally tailored stiffness, as, e.g., required for bone implants that mimic the strong spatial variations in the stiffness of natural bone (63). UCs may vary in the shape of their external bounding box (due to the applied affine transformations) and their internal topology, both of which must be considered when introducing spatial gradings between UCs. Without the introduced rotations and affine transformations, a smooth transition from one topology to another could be achieved by grading the diameters of the struts in the UCs (23, 41, 49, 64), since all topologies have the same cube bounding box and connectivities to the corner vertices. Here, by contrast, smooth transitions between affinely transformed UCs (which maintain their connectivity but alter the bounding box) are challenging. We leverage the fact that all predicted UC bounding boxes are trapezoidal primitive cells spanned by three translation vectors $\left\{a_{1},a_{2},a_{3}\right\}$ (Fig. 3*B*). We transform these—spatially varying—translation vectors in a graded structure into their reciprocal lattice space, where we perform the required spatial interpolation, before converting this interpolated representation back to real space. This results in a spatially graded structure, which smoothly interpolates between the given primitive cells or, in our case, between different affine transformations of the formerly cubic UCs (for details see *SI Appendix*, section 5). In addition, we perform the aforementioned smooth grading of strut diameters to connect dissimilar UC topologies. An example is illustrated in Fig. 3*B*, which smoothly transitions between two UCs previously predicted to match bone stiffnesses (Fig. 3*A*). This approach produces graded truss networks, whose effective anisotropic stiffness varies from point to point and can be tuned to match known stiffnesses at control points (e.g., anatomical sites in bone samples with measured stiffness). The spatial grading of UCs can be applied more generally to functionally graded structures, optimized, e.g., for the response to known loads (such as in multiscale topology optimization) (31) and, by locally tailoring wave motion by lattice topology, for wave guidance (6) and acoustic cloaking (65).

## Materials and Methods

Details of the generated lattice design catalog (*SI Appendix*, section 1); the computational homogenization scheme (*SI Appendix*, section 2); the machine-learning framework, training protocols, and accuracy (*SI Appendix*, section 3); the evaluation on stiffnesses outside the proposed design space (*SI Appendix*, section 4); and the method to create spatially graded lattices (*SI Appendix*, section 5) are provided in *SI Appendix*.

## Supplementary Material

Supplementary File

## Data Availability

The code used to train the inverse design framework and obtain predictions has been uploaded to GitHub (https://github.com/jhbastek/InvertibleTrussDesign) (66). The corresponding training data has been deposited in the ETHZ Research Collection (https://doi.org/10.3929/ethz-b-000520254) (67).

## Footnotes

*By topology we refer to a class of (infinitely many) UCs that can be continuously transformed into each other by stretching or rotation (as discussed below). Two UCs have the same topology if they have the same elementary lattices and respective tessellations.

^†^The stiffness of a strut in stretching vs. bending scales differently with the strut thickness (namely, with the cross-sectional area $A\propto d^{2}$ vs. with the area moment of inertia $I\propto d^{4}$, respectively). Therefore, the UC response to straining depends considerably and nonlinearly on the strut thickness.
