# Supplementary material for: Inverting the structure–property map of truss metamaterials by deep learning
Source: Proc Natl Acad Sci U S A. 2021 Dec 30;119(1):e2111505119. doi: 10.1073/pnas.2111505119 (PMC8740766; doi:10.1073/pnas.2111505119)
Supplement: Supplementary File [file pnas.2111505119.sapp.pdf]

PNAS
www.pnas.org

Supporting Information for

Inverting the structure-property map of truss metamaterials by deep learning

Jan-Hendrik Bastek, Siddhant Kumar, Bastian Telgen, Raphaël N. Glaesener and Dennis M. Kochmann

Correspondence to: Dennis M. Kochmann (dmk@ethz.ch)

This PDF file includes:

Supplementary text
Figs. S1 to S8
Tables S1 to S12
SI References

Supporting Information Text

1. Details on the lattice design space and dataset generation

Fig. S1 shows the seven considered elementary topologies in the proposed lattice design space. Each row of matrix $\boldsymbol{X}$ corresponds to the 3D positional coordinates of a truss junction (or *node*) in the UC, while matrix $\boldsymbol{C}$ defines the connectivities between truss junctions (being 1-indexed, each row defines a connection between the nodes with the given two indices).

The full set of parameters describing the design space of our truss lattices is summarized in Table S1. A dataset of 3 million lattices with their corresponding homogenized stiffness response was generated by drawing with a (discrete) uniform distribution within each of the provided ranges; e.g., the relative density is randomly sampled as $\rho \sim U(0.002, 0.1)$. For the rotations, the angle-axis representation was used, where an angle $\theta \sim U(-\pi, \pi)$ and a random unit vector (as the axis of rotation) on the three-dimensional unit sphere $S^2 = \{\boldsymbol{k} \in \mathbb{R}^3 : \|\boldsymbol{k}\| = 1\}$ was drawn.

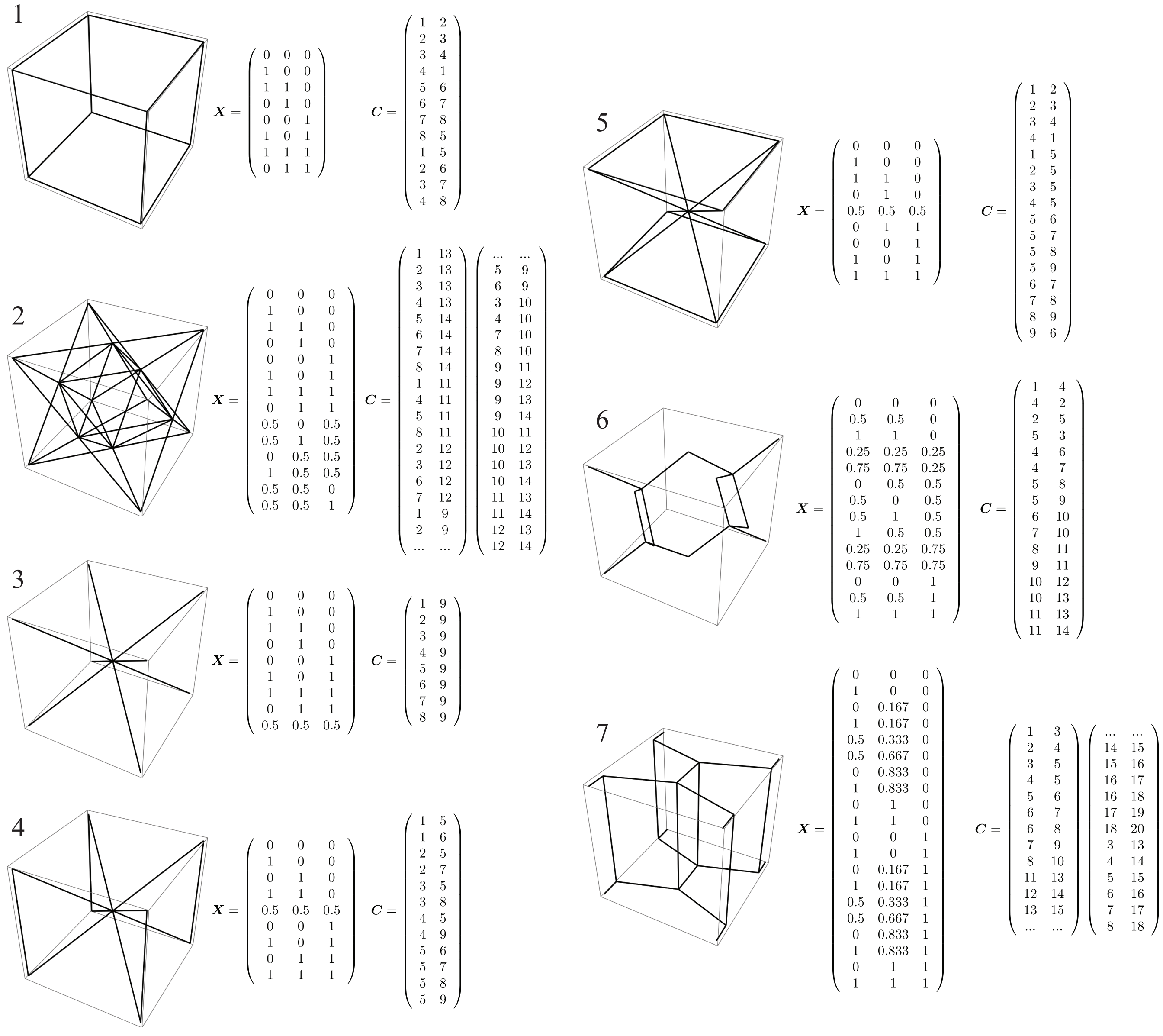

Fig. S1. The seven considered elementary truss UC topologies along with their nodal positions $\boldsymbol{X}$ and connectivities $\boldsymbol{C}$.

2. Details on computational homogenization and stiffness visualization

To extract the homogenized stiffness tensor components $\mathbb{C}_{ijkl}$ from a UC in our design space, we used the finite element method. We modeled each strut in the UC as a linear elastic Timoshenko beam element with circular cross-section. Any beam intersections, arising for certain combinations of elementary cells and tessellations, were resolved by introducing a new vertex at each intersection point and subsequently splitting the affected struts into adjoining sub-struts. We imposed periodic boundary conditions onto the boundary nodes of the UC (i.e., periodic displacement and anti-periodic force boundary conditions applied to all nodes on the outer UC boundary, while boundary rotations were set to be periodic). The effective stiffness tensor components $\mathbb{C}_{ijkl}$ were extracted from the resulting assembled UC stiffness matrix, following (1).

To visualize the elastic surfaces, we plot the effective Young's modulus along a direction $\boldsymbol{d} \in S^2$ ($S^2 = \{\boldsymbol{k} \in \mathbb{R}^3 : \|\boldsymbol{k}\| = 1\}$ denoting the unit sphere in 3D) as

$$E(\boldsymbol{d}) = \left(\sum_{i,j,k,l=1}^{3} \mathbb{C}^{-1}_{ijkl} d_i d_j d_k d_l \right)^{-1} . \qquad [1]$$

3. Machine learning framework

A. Data preparation. The continuous (geometrical) features, consisting of the relative density ρ and the six stretch tensor eigenvalues U_1, U_2, U_3 and V_1, V_2, V_3, were rescaled with a min-max normalization to the range $[0, 1]$, i.e.,

$$\rho \leftarrow \frac{\rho - 0.002}{0.1 - 0.002}, \quad U_i \leftarrow \frac{U_i - 0.5}{2.0 - 0.5}, \quad V_i \leftarrow \frac{V_i - 0.5}{2.0 - 0.5}, \quad i = 1, 2, 3. \qquad [2]$$

The categorical (topological) features, consisting of the first six parameters in Table S1, were one-hot encoded to prevent an ordinal interpretation of the different topologies, while the rotations $\boldsymbol{R}_I$, $\boldsymbol{R}_{II}$ (which form a part of the geometrical features) were left unmodified, as they directly enter the manually computed tensor rotations. The stiffness labels were consistently scaled with a min-max normalization to the range $[-1, 1]$, i.e.,

$$\mathbb{C}_i \leftarrow \frac{2\,[\mathbb{C}_i - \min(\mathbb{C}_i)]}{\max(\mathbb{C}_i) - \min(\mathbb{C}_i)} - 1, \qquad [3]$$

where $\mathbb{C}_i$ stands for each of the 21 independent stiffness constants, and each $\mathbb{C}_i$ was treated independently to enforce equal contributions to the loss of the 9 (for $\mathcal{F}_1$) or 21 (for $\mathcal{F}_2$ and $\mathcal{G}_1$, $\mathcal{G}_2$) given stiffness parameters during training. Each stiffness parameter is then scaled to its original value via

$$\mathbb{C}_i \leftarrow 0.5\,(\mathbb{C}_i + 1)\;[\max(\mathbb{C}_i) - \min(\mathbb{C}_i)] + \min(\mathbb{C}_i), \qquad [4]$$

before applying rotations at the intermediate stages within the forward model to correctly evaluate the rotation.

Table S1. Overview of the lattice design space described by both discrete and continuous design parameters summarized in vector $\boldsymbol{\Theta}$. Rotations $\boldsymbol{R}_I$ and $\boldsymbol{R}_{II}$ can each be represented by three parameters such as, e.g., via the angle-axis representation, which was used here to generate the dataset. Note that each elementary topology is used as a single tessellation ($t_i = 1$) or a $2 \times 2 \times 2$ tesselation ($t_i = 2$), which is binary-encoded.

Design parameters $\boldsymbol{\Theta}$	Range
First elementary topology S_1	$\{1, 2, 3, 4, 5, 6, 7\}$
Tesselation of first topology t_1	$\{1, 2\}$
Second elementary topology S_2	$\{1, 2, 3, 4, 5, 6, 7\}$
Tesselation of second topology t_2	$\{1, 2\}$
Third elementary topology S_3	$\{1, 2, 3, 4, 5, 6, 7\}$
Tesselation of third topology t_3	$\{1, 2\}$
Relative density ρ	$[0.002, 0.1]$
Eigenvalues of the first stretch tensor U_1, U_2, U_3	$[0.5, 2.0]$
Eigenvalues of the second stretch tensor V_1, V_2, V_3	$[0.5, 2.0]$
Rotations $\boldsymbol{R}_I$, $\boldsymbol{R}_{II}$	SO(3)

B. Details of the inverse model and tensor rotations. The inverse model has different activation functions applied to its outputs in the final layer, depending on the type of design parameter the output is corresponding to. For the one-hot encoded discrete predictions (relating to the topology of the proposed lattice), the *Gumbel-softmax* trick (corresponding to Eqs. (5) and (6) of the main article) is applied. For the geometrical predictions relating to ρ, U_1, U_2, U_3 and V_1, V_2, V_3, the sigmoid activation function

$$\text{sig}(\boldsymbol{\Theta}) = \frac{1}{1 + \exp(-\boldsymbol{\Theta})} \tag{5}$$

is applied to ensure that predicted values are within the same (min-max-normalized) range of values provided within our training data. To obtain the two rotation matrices $\boldsymbol{R}_I$ and $\boldsymbol{R}_{II}$, we leverage that any rotation matrix $\boldsymbol{R} \in \text{SO}(3)$ can be constructed in a Gram-Schmidt-like process via

$$\boldsymbol{R} = \begin{bmatrix} | & | & | \\ b_1 & b_2 & b_3 \\ | & | & | \end{bmatrix} \quad \text{with} \quad b_i = \left[\begin{Bmatrix} N(a_1) & \text{if } i = 1 \\ N(a_2 - (b_1 \cdot a_2)b_1) & \text{if } i = 2 \\ b_1 \times b_2 & \text{if } i = 3 \end{Bmatrix}\right]^T, \tag{6}$$

where $N(\cdot)$ represents a normalization function, and $a_1, a_2 \in \mathbb{R}^3$ correspond to the so-called *6D representation* (2). These six parameters are directly assembled by the output of our inverse model (i.e., no activation function is applied) and, since we sample two rotation matrices, this results in a total of 12 additional output dimensions characterizing the rotations. Most importantly, it can be shown that this rotation parameterization is a continuous representation of the space of SO(3) rotations (unlike Euler angles or quaternions), which facilitates the training of NNs (2). Although we did not conduct a rigorous comparison of the NN performance for different rotation representations, results indicated a small improvement in the accuracy of the inverse prediction using the above 6D parameterization compared to other representations. All stiffness tensor rotations were computed by using Voigt notation, i.e., considering $\mathbb{C}$ in Voigt notation and applying the transformation

$$\hat{\mathbb{C}} = \boldsymbol{K} \, \mathbb{C} \, \boldsymbol{K}^T, \tag{7}$$

where

$$\boldsymbol{K} = \left[\begin{array}{c|c} \boldsymbol{K}_1 & 2\boldsymbol{K}_2 \\ \hline \boldsymbol{K}_3 & \boldsymbol{K}_4 \end{array}\right], \tag{8}$$

with submatrices

$$\boldsymbol{K}_1 = \begin{bmatrix} R_{11}^2 & R_{12}^2 & R_{13}^2 \\ R_{21}^2 & R_{22}^2 & R_{23}^2 \\ R_{31}^2 & R_{32}^2 & R_{33}^2 \end{bmatrix}, \quad \boldsymbol{K}_2 = \begin{bmatrix} R_{12}R_{13} & R_{13}R_{11} & R_{11}R_{12} \\ R_{22}R_{23} & R_{23}R_{21} & R_{21}R_{22} \\ R_{32}R_{33} & R_{33}R_{31} & R_{31}R_{32} \end{bmatrix}, \quad \boldsymbol{K}_3 = \begin{bmatrix} R_{21}R_{31} & R_{22}R_{32} & R_{23}R_{33} \\ R_{31}R_{11} & R_{32}R_{12} & R_{33}R_{13} \\ R_{11}R_{21} & R_{12}R_{22} & R_{13}R_{23} \end{bmatrix},$$
$$\boldsymbol{K}_4 = \begin{bmatrix} R_{22}R_{33} + R_{23}R_{32} & R_{21}R_{33} + R_{23}R_{31} & R_{22}R_{31} + R_{21}R_{32} \\ R_{12}R_{33} + R_{13}R_{32} & R_{13}R_{31} + R_{11}R_{33} & R_{11}R_{32} + R_{12}R_{31} \\ R_{12}R_{23} + R_{13}R_{22} & R_{13}R_{21} + R_{11}R_{23} & R_{11}R_{22} + R_{12}R_{21} \end{bmatrix}, \tag{9}$$

where R_{ij} refer to the components of the second-order rotation tensor. This formulation bypasses transformations between the Voigt stiffness matrix and the fourth-order stiffness tensor and was first described by Bond (3). Predicted geometrical design features $\boldsymbol{\Theta}_i^{pred}$ were rescaled to the corresponding physical ranges (see Table S1), e.g., for the relative density via

$$\rho^{pred} \leftarrow \rho^{pred} \, (0.1 - 0.002) + 0.002, \tag{10}$$

during post-processing and FEM reconstruction.

C. Protocols for NN training. Details of the architectures and other training parameters for both NNs of the forward model ($\mathcal{F}_1$ and $\mathcal{F}_2$) and the inverse model ($\mathcal{G}_1$ and $\mathcal{G}_2$ share the same hyperparameters) are provided in Table S2. We used 1% of the generated dataset for the optimization of the presented hyperparameters.

Hidden layers perform a linear transformation followed by a leaky rectified linear unit (leaky ReLU)

$$f(x) = \begin{cases} 0.01x & \text{for} \quad x < 0 \\ x & \text{for} \quad x \geq 0 \end{cases} \tag{11}$$

as an activation function. The PyTorch package (4) was used throughout our implementation, which also allows for automatic differentiation and thus straightforward evaluation of the required gradients of the tensor rotations (Eq. (7)) during training via its automatic differentiation engine, autograd. Note that the learning rate was dynamically reduced during training of the inverse model, using PyTorch's `ReduceLROnPlateau` scheduler with a patience of 20 and reduction factor of 0.5. Network trainings were performed on a single Nvidia Quadro RTX 6000 with 24 GB GDDR6 memory.

Fig. S2 show the loss of all considered NNs at every training epoch (the two NNs of the forward model, and the inverse model whose NNs were trained jointly). Train and test loss behave similarly, indicating no significant overfitting.

Table S2. Input and output dimensions and hyperparameters for the optimized forward and inverse models. The feature scaling for the second NN of the forward model, $\mathcal{F}_2$, is only applied to the stretch eigenvalues V_1, V_2, V_3; the rotated stiffness given as an input is left unchanged. Feature and label scalings are only applied to continuous design parameters (excluding rotations). [1]Learning rate decay was applied using PyTorch's `ReduceLROnPlateauscheduler` with a patience of 20 and reduction factor of 0.5.

	Forward model ($\mathcal{F}_1$)	Forward model ($\mathcal{F}_2$)	Inverse model ($\mathcal{G}_1, \mathcal{G}_2$)
Input dimension	31	24	21
Hidden layer dimensions	$512, 512$	$1024, 1024, 1024$	$2560, 2560, 1280, 1280, 640, 640$
Ouput dimension	9	21	46
Activation functions	Leaky ReLU	Leaky ReLU	Leaky ReLU
Feature scaling	min-max (see Eq. (2))	none, min-max (see Eq. (2))	min-max (see Eq. (3))
Label scaling	min-max (see Eq. (3))	min-max (see Eq. (3))	min-max (see Eq. (3))
Optimization algorithm	Adam (5)	Adam (5)	Adam (5)
Learning rate	0.001	0.001	0.001 (decaying[1])
Batch size	8192	8192	8192
Number of epochs	100	150	250
Dropout	none	none	none

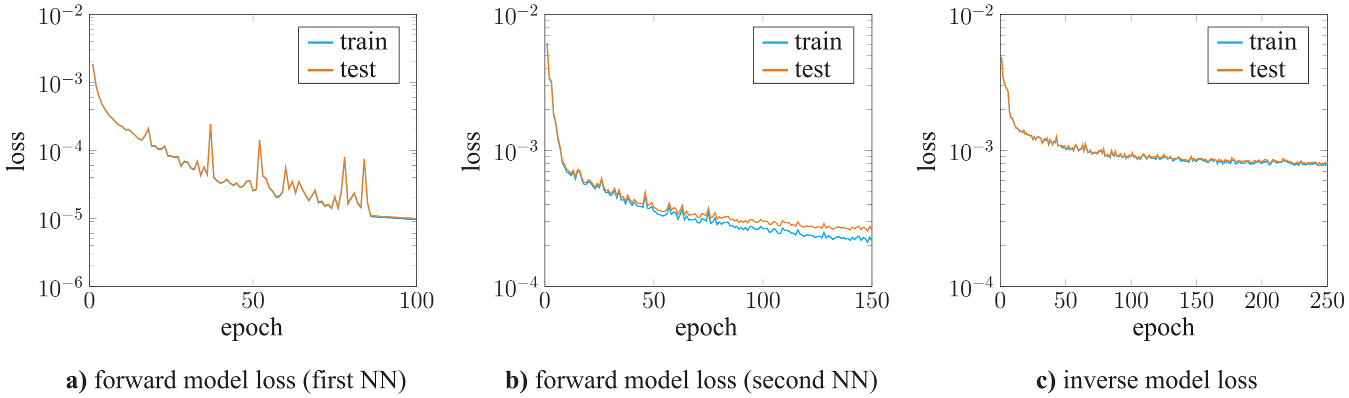

Fig. S2. Loss plots for a) the first NN of the forward model ($\mathcal{F}_1$), b) the second NN of the forward model ($\mathcal{F}_2$), and c) the inverse model ($\mathcal{G}$). Both NNs of the forward model are evaluated with FEM, while the reconstruction accuracy of the inverse model is evaluated with the trained forward model.

D. Evaluation of accuracy. In the main article, the $\mathbb{C}_{1111}$ reconstruction accuracy of both the forward and inverse model was shown (Figure 2). The full reconstruction accuracy of all stiffness components (including R^2-values) for the forward and inverse models is summarized in Figures S3 and Fig. S4, respectively. All metrics of accuracy are evaluated on a separate test dataset (previously unseen during training) comprised of 30,000 lattices and their corresponding stiffness components.

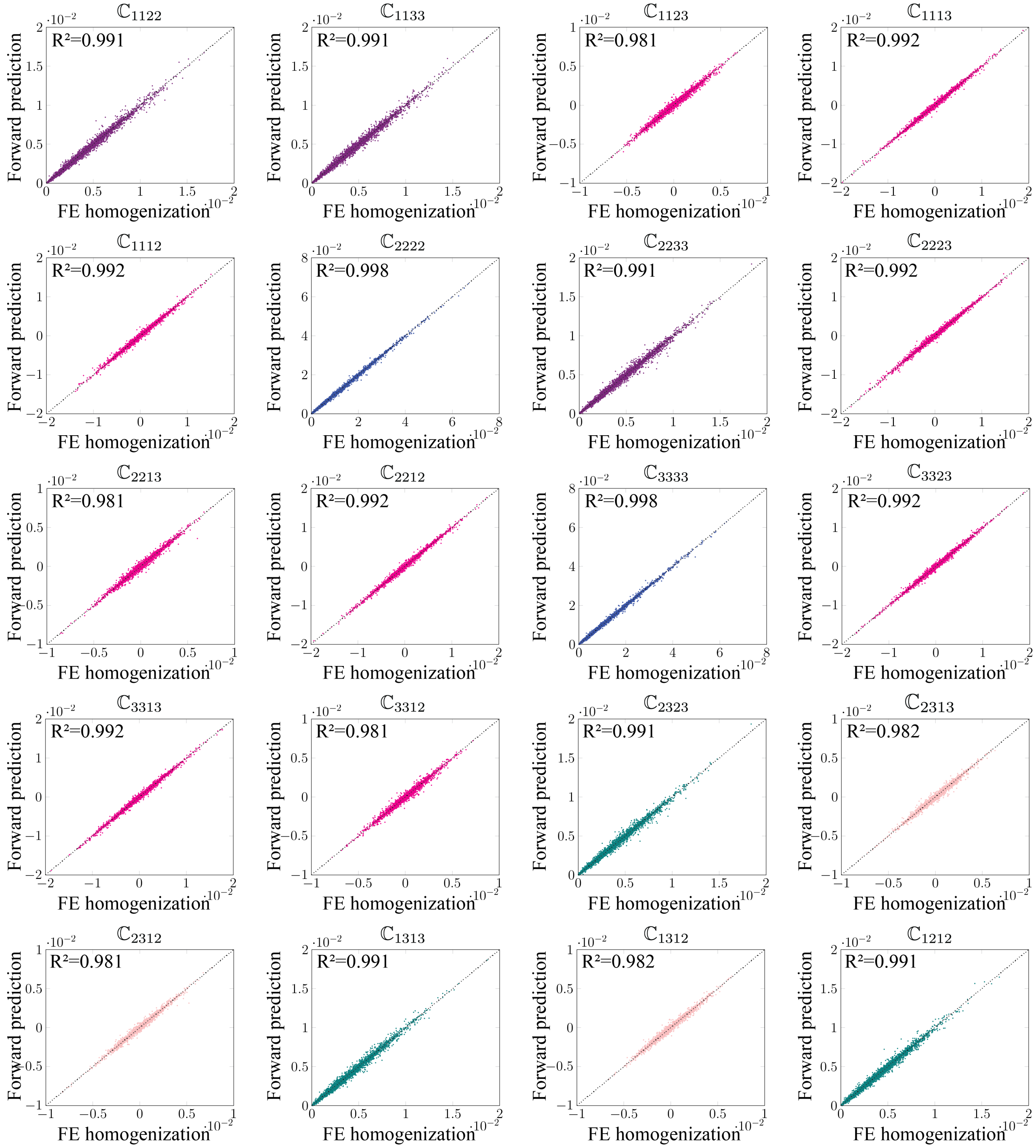

Fig. S3. Comparison of the stiffness of the drawn lattices evaluated via FE homogenization with the predicted stiffness from the forward model over the test set ($\mathbb{C}_{1111}$ is excluded here since it is indicated in the main article). The corresponding R^2-deviations are indicated. Different colors indicate the different coupling terms of the elasticity tensor (i.e., pure extension/compression: blue, normal/normal: violet, normal/shear: magenta, pure shear: green, shear/shear: pink).

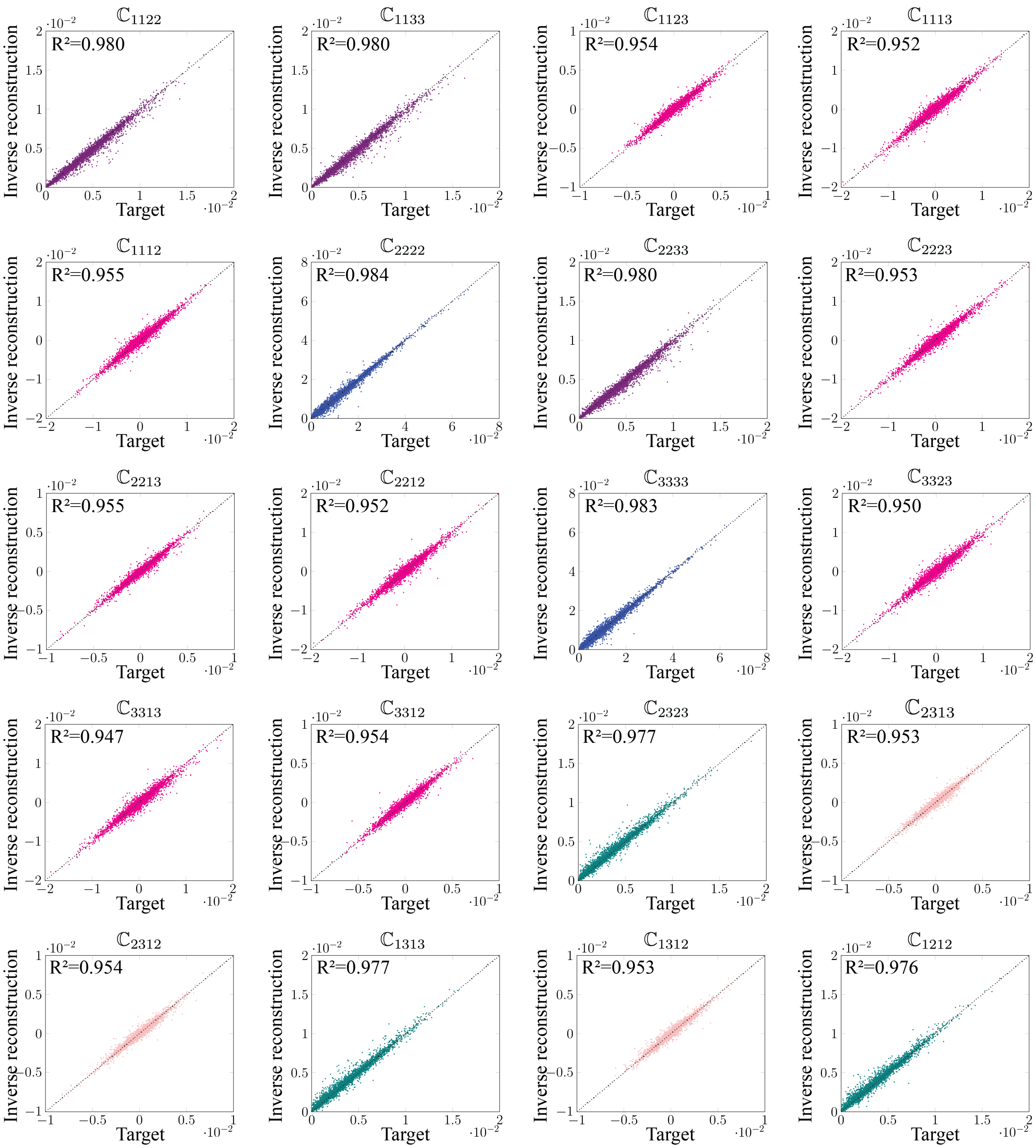

Fig. S4. Comparison of the target stiffness with the reconstructed (via forward model) stiffness of the lattice proposed by the inverse model over the test set ($\mathbb{C}_{1111}$ is excluded here since it is indicated in the main article). The corresponding R^2-deviations are indicated. Note that due to the stochastic nature of our inverse model, R^2-deviations can vary for different drawings; however, these differences are negligible for the given test set. Different colors indicate the different coupling terms of the elasticity tensor (i.e., pure extension/compression: blue, normal/normal: violet, normal/shear: magenta, pure shear: green, shear/shear: pink).

E. Variational sampling. To showcase the generative nature of our inverse variational model, Figure S5 presents the obtained inverse predictions for four randomly selected lattices in the test set (labeled A, B, C, and D). Except for lattice B, we obtain different topologies for a different second seed. Note that we can, however, trade off some reconstruction accuracy for a larger topology variation by increasing τ in our softmax layer (see Eq. (5) of the main article), as this increasingly flattens the posterior probability distribution of the predicted topologies. While setting τ too large will drastically deteriorate the results, we noticed that a moderate raise does increase the variance without a notable loss in accuracy. The design parameters for each of the cases A, B, C, and D are given, respectively, in Tables S3, S4, S5, and S6.

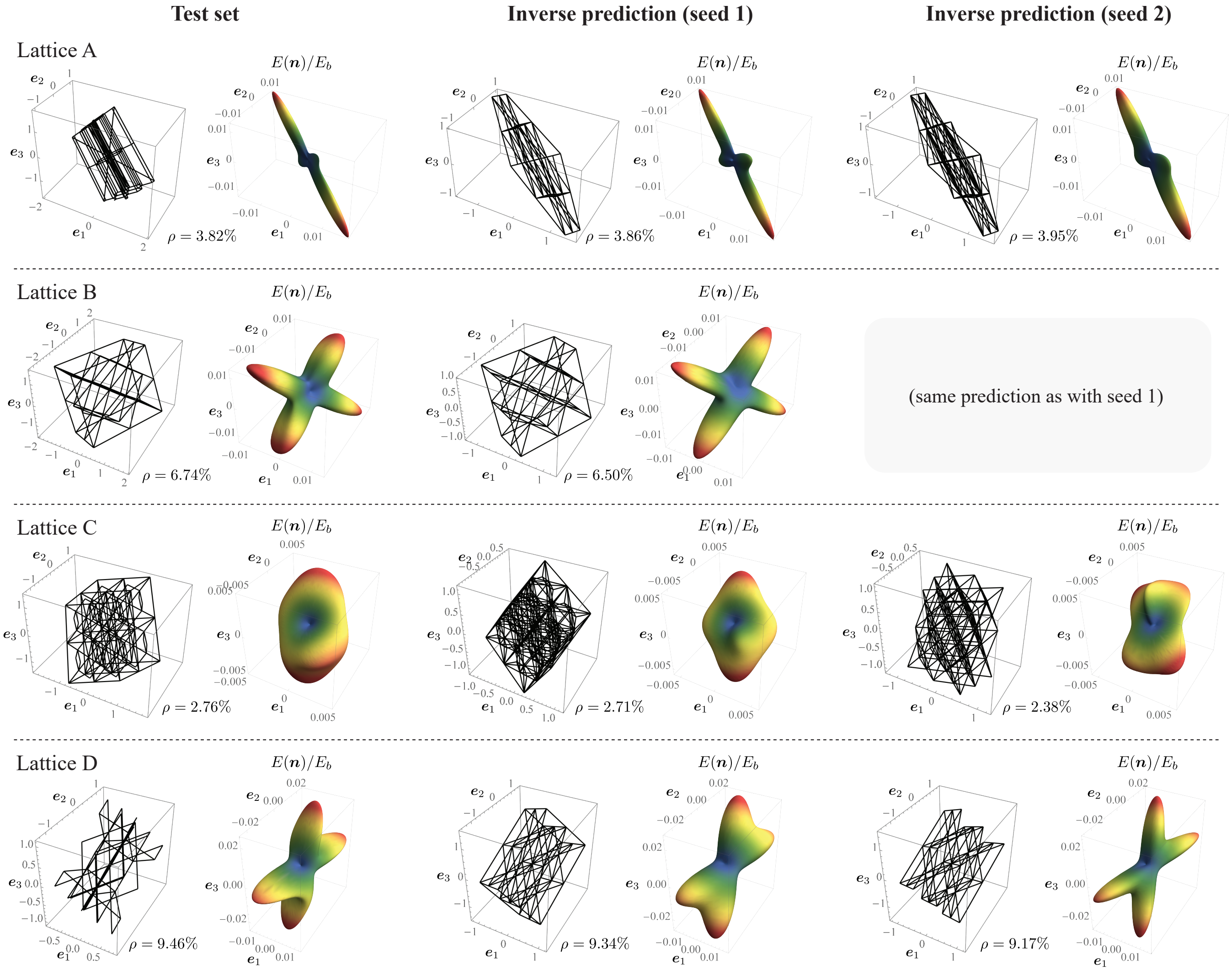

Fig. S5. Selection of design queries of the test set and predicted lattice candidates, including their (normalized) Young's Modulus $E(\boldsymbol{n})$ for all directions $\boldsymbol{n} \in S^2$ in the Cartesian basis $\{\boldsymbol{e}_1, \boldsymbol{e}_2, \boldsymbol{e}_3\}$ for different random seeds.

 Jan-Hendrik Bastek, Siddhant Kumar, Bastian Telgen, Raphaël N. Glaesener and Dennis M. Kochmann

Table S3. Overview of the inverse-designed lattice parameters Θ^{pred}, given the stiffness of lattice A from the test set. Rotations R_I and R_{II} are given in angle-axis representation, where θ indicates the rotation angle about the normalized rotation axis (e_1, e_2, e_3) with $e_3 = \sqrt{1 - e_1^2 - e_2^2}$.

Design parameters $\mathbf{\Theta}$	Lattice A (test set)	Inverse prediction (seed 1)	Inverse prediction (seed 2)
First elementary topology	4	1	2
Tesselation of first topology	1	1	1
Second elementary topology	7	3	3
Tesselation of second topology	2	2	2
Third elementary topology	7	5	5
Tesselation of third topology	2	2	2
Relative density ρ	3.82%	3.86%	3.95%
Stretches U_1, U_2, U_3	$\{1.41, 0.92, 1.72\}$	$\{1.77, 0.58, 1.38\}$	$\{1.73, 0.59, 1.43\}$
Stretches V_1, V_2, V_3	$\{0.541.82, 1.50\}$	$\{0.73, 1.95, 0.67\}$	$\{0.75, 1.95, 0.70\}$
Rotation $\boldsymbol{R}_I$ $\{\theta, e_1, e_2\}$	$\{2.58, 0.91, -0.41\}$	$\{2.60, 0.02, 0.66\}$	$\{2.60, 0.04, 0.66\}$
Rotation $\boldsymbol{R}_{II}$ $\{\theta, e_1, e_2\}$	$\{-2.66, -0.83, -0.56\}$	$\{2.30, -0.37, 0.82\}$	$\{2.33, -0.36, 0.82\}$

Table S4. Overview of the inverse-designed lattice parameters Θ^{pred}, given the stiffness of lattice B from the test set. Rotations R_I and R_{II} are given in angle-axis representation, where θ indicates the rotation angle about the normalized rotation axis (e_1, e_2, e_3) with $e_3 = \sqrt{1 - e_1^2 - e_2^2}$.

Design parameters $\mathbf{\Theta}$	Lattice B (test set)	Inverse prediction (seed 1 & 2)
First elementary topology	1	1
Tesselation of first topology	1	1
Second elementary topology	3	3
Tesselation of second topology	1	2
Third elementary topology	3	4
Tesselation of third topology	2	2
Relative density ρ	6.74%	6.50%
Stretch U_1, U_2, U_3	$\{0.93, 1.91, 1.78\}$	$\{1.55, 1.65, 0.76\}$
Stretch V_1, V_2, V_3	$\{1.77, 1.99, 1.46\}$	$\{1.45, 1.58, 1.09\}$
Rotation $\boldsymbol{R}_I$ $\{\theta, e_1, e_2\}$	$\{-1.66, -0.9, 0.42\}$	$\{1.27, -0.39, 0.57\}$
Rotation $\boldsymbol{R}_{II}$ $\{\theta, e_1, e_2\}$	$\{0.07, -0.74, -0.04\}$	$\{-2.75, -0.28, 0.95\}$

Table S5. Overview of the inverse-designed lattice parameters Θ^{pred}, given the stiffness of lattice C from the test set. Rotations R_I and R_{II} are given in angle-axis representation, where θ indicates the rotation angle about the normalized rotation axis (e_1, e_2, e_3) with $e_3 = \sqrt{1 - e_1^2 - e_2^2}$.

Design parameters $\mathbf{\Theta}$	Lattice C (test set)	Inverse prediction (seed 1)	Inverse prediction (seed 2)
First elementary topology	1	1	2
Tesselation of first topology	2	2	1
Second elementary topology	4	2	2
Tesselation of second topology	2	2	2
Third elementary topology	6	6	6
Tesselation of third topology	2	2	1
Relative density ρ	2.76%	2.71%	2.38%
Stretch U_1, U_2, U_3	$\{1.37, 0.96, 1.36\}$	$\{1.41, 1.10, 1.08\}$	$\{1.25, 1.33, 0.97\}$
Stretch V_1, V_2, V_3	$\{1.91, 1.92, 0.85\}$	$\{1.27, 1.65, 0.62\}$	$\{1.32, 1.62, 0.64\}$
Rotation $\boldsymbol{R}_I$ $\{\theta, e_1, e_2\}$	$\{-1.81, 0.81, 0.45\}$	$\{2.24, -0.23, 0.85\}$	$\{2.35, -0.26, 0.90\}$
Rotation $\boldsymbol{R}_{II}$ $\{\theta, e_1, e_2\}$	$\{0.82, 0.84, -0.23\}$	$\{2.85, -0.23, 0.85\}$	$\{2.67, -0.41, 0.85\}$

Table S6. Overview of the inverse-designed lattice parameters Θ^{pred}, given the stiffness of lattice D from the test set. Rotations $\boldsymbol{R}_I$ and $\boldsymbol{R}_{II}$ are given in angle-axis representation, where θ indicates the rotation angle about the normalized rotation axis (e_1, e_2, e_3) with $e_3 = \sqrt{1 - e_1^2 - e_2^2}$.

Design parameters $\boldsymbol{\Theta}$	Lattice D (test set)	Inverse prediction (seed 1)	Inverse prediction (seed 2)
First elementary topology	3	1	5
Tesselation of first topology	2	1	1
Second elementary topology	6	3	5
Tesselation of second topology	1	1	2
Third elementary topology	7	5	6
Tesselation of third topology	1	2	1
Relative density ρ	9.46%	9.34%	9.17%
Stretch U_1, U_2, U_3	$\{0.86, 1.33, 0.54\}$	$\{1.29, 1.72, 0.99\}$	$\{1.33, 1.74, 0.98\}$
Stretch V_1, V_2, V_3	$\{1.06, 1.96, 1.60\}$	$\{1.46, 1.65, 0.86\}$	$\{1.48, 1.68, 0.84\}$
Rotation $\boldsymbol{R}_I$ $\{\theta, e_1, e_2\}$	$\{2.19, 0.98, 0.08\}$	$\{1.97, -0.42, 0.83\}$	$\{2.03, -0.52, 0.72\}$
Rotation $\boldsymbol{R}_{II}$ $\{\theta, e_1, e_2\}$	$\{1.96, 0.54, -0.84\}$	$\{-2.87, -0.42, 0.80\}$	$\{-3.01, -0.54, 0.73\}$

F. Effect of the dataset size. Large datasets (such as the chosen one containing 3 million lattices and their corresponding stiffnesses) may be expensive to generate in other scenarios, which is why we assessed the performance of our framework on a smaller dataset as well. We re-trained the networks using only 10% of the lattice-stiffness pairs of the original dataset and provide the resulting loss plots in Figure S6. For the forward model, the decrease in the corresponding coefficient of determination R^2 is between 0.004 (for the more dominant terms such as $\mathbb{C}_{1111}$) and 0.031 (for the less dominant terms such as $\mathbb{C}_{1312}$), as shown in detail in Table S7. The inverse model shows a similar behavior, the decrease in R^2 ranges from around 0.01 (for $\mathbb{C}_{1111}$) to 0.054 (for $\mathbb{C}_{1212}$). As expected, the models perform slightly worse than those trained on the significantly larger dataset. However, the R^2 values indicate that the accuracy has not deteriorated drastically. We therefore conclude that the framework still performs comparably well, if only a fraction of the data is provided (which is of importance when data generation is a limiting factor). Note that, as a benefit of our physics-informed approach, data augmentation by adding random rotations of the provided lattices can easily extend the dataset for inverse training without requiring additional FEM simulations (but this was not performed in this work).

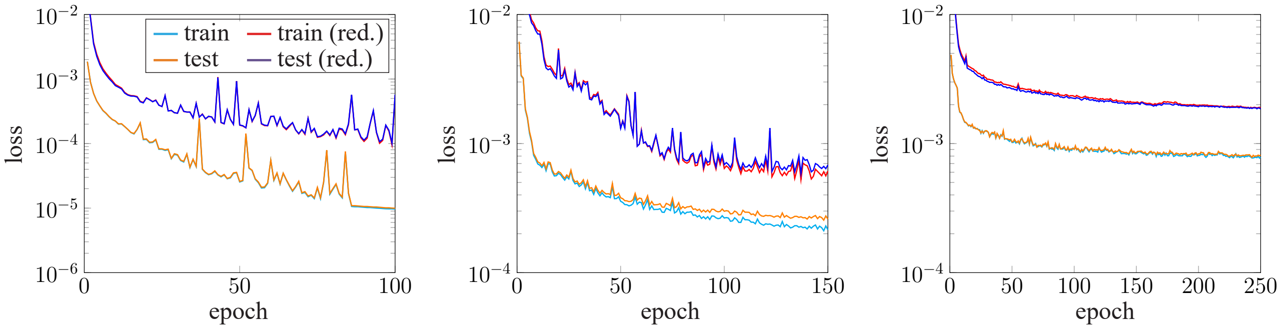

a) forward model loss (first NN) **b)** forward model loss (second NN) **c)** inverse model loss

Fig. S6. Loss plots for **a)** the first NN of the forward model ($\mathcal{F}_1$), **b)** the second NN of the forward model ($\mathcal{F}_2$), and **c)** the inverse model ($\mathcal{G}$) by providing the original dataset consisting of $3{,}000{,}000$ and a reduced dataset consisting of $300{,}000$ lattices and their respective stiffnesses, denoted by *train/test* and *train (red.)/test (red.)*, respectively.

Table S7. Overview of the R^2-deviations of the forward and inverse model for the full and reduced datasets (containing $3{,}000{,}000$ and $300{,}000$ lattice-stiffness pairs, respectively). The forward models are evaluated with FEM, while the reconstruction accuracies of the inverse models are evaluated with the forward model (trained on the corresponding dataset).

Elastic const.	Fwd. model (full)	Fwd. model (red.)	Inv. model (full)	Inv. model (red.)
$\mathbb{C}_{1111}$	0.998	0.994	0.986	0.976
$\mathbb{C}_{1122}$	0.991	0.976	0.980	0.940
$\mathbb{C}_{1133}$	0.991	0.976	0.980	0.933
$\mathbb{C}_{1123}$	0.981	0.954	0.954	0.909
$\mathbb{C}_{1113}$	0.992	0.984	0.952	0.917
$\mathbb{C}_{1112}$	0.992	0.982	0.955	0.914
$\mathbb{C}_{2222}$	0.998	0.994	0.984	0.973
$\mathbb{C}_{2233}$	0.991	0.977	0.980	0.938
$\mathbb{C}_{2223}$	0.992	0.984	0.953	0.923
$\mathbb{C}_{2213}$	0.981	0.952	0.955	0.915
$\mathbb{C}_{2212}$	0.992	0.984	0.952	0.912
$\mathbb{C}_{3333}$	0.998	0.994	0.983	0.970
$\mathbb{C}_{3323}$	0.992	0.982	0.950	0.901
$\mathbb{C}_{3313}$	0.992	0.983	0.947	0.913
$\mathbb{C}_{3312}$	0.981	0.952	0.954	0.912
$\mathbb{C}_{2323}$	0.991	0.977	0.977	0.928
$\mathbb{C}_{2313}$	0.982	0.954	0.953	0.913
$\mathbb{C}_{2312}$	0.981	0.952	0.954	0.908
$\mathbb{C}_{1313}$	0.991	0.974	0.977	0.930
$\mathbb{C}_{1312}$	0.982	0.951	0.953	0.912
$\mathbb{C}_{1212}$	0.991	0.975	0.976	0.922

G. Performance of the inverse model compared to the best match in the training data. As the dataset we used to train the networks contains 3 million lattice-stiffness pairs, it may seem like such a large dataset should directly contain (close to) optimal designs for a given stiffness. However, the curse of dimensionality renders these 3 million samples negligible to the total number of possible combinations. Consider a very coarse sampling of the design space, e.g., taking only 10 different numerical choices for each of the 13 continuous (geometric) design parameters. Given the 262 chosen truss topologies, this gives 262×10^{13} possible combinations. Generating such large datasets and comparing them to a given target stiffness is computationally expensive – prohibitively expensive for applications requiring repeated inverse predictions (such as, e.g., for two-scale topology optimization). To be specific, finding the closest match in our given database for a given stiffness using the Pandas library for Python (6) took on average ca. 35s (performed on an Intel Xeon Silver 4114 2.2GHz processor), whereas our inverse model predicts a design within ca. 2ms (30,000 stiffnesses are evaluated in about 60s on the same machine). This four-orders-of-magnitude difference in efficiency is a clear advantage over the purely data-driven lookup tables approach. Besides, even if a dataset of 3 million entries could be sufficient – how would one choose the 3 million samples from the (at least) 262×10^{13} possible combinations to cover a sufficient stiffness space? Our approach, which interpolates (in the latent space) between the chosen 3 million samples, indeed provides a sufficiently rich stiffness space. Notably, we have shown in Section 3.F that the use of a reduced dataset for training (e.g., only 10% of the original dataset of 3 million lattice-stiffness pairs) still achieves good accuracy in inverse design prediction.

In addition, let us demonstrate that our framework generates better designs as compared to the closest match from the training dataset. To give a specific example, we identified the closest match in our database for the *hex_Z04._R145* sample from the crystallographic periodic network database (7) (presented in Figure S7a). This architecture is not contained in our design space (it is not even close to the chosen elementary topologies). We identified the best match from our training set by minimizing the squared error for all 21 elastic components, and in Figure S7 we compare it to the reported prediction of our inverse model. For this example, the inverse-model prediction is much closer to the given target stiffness (normalized mean squared error of 0.003) than the best match from the training dataset (normalized mean squared error of 0.036).

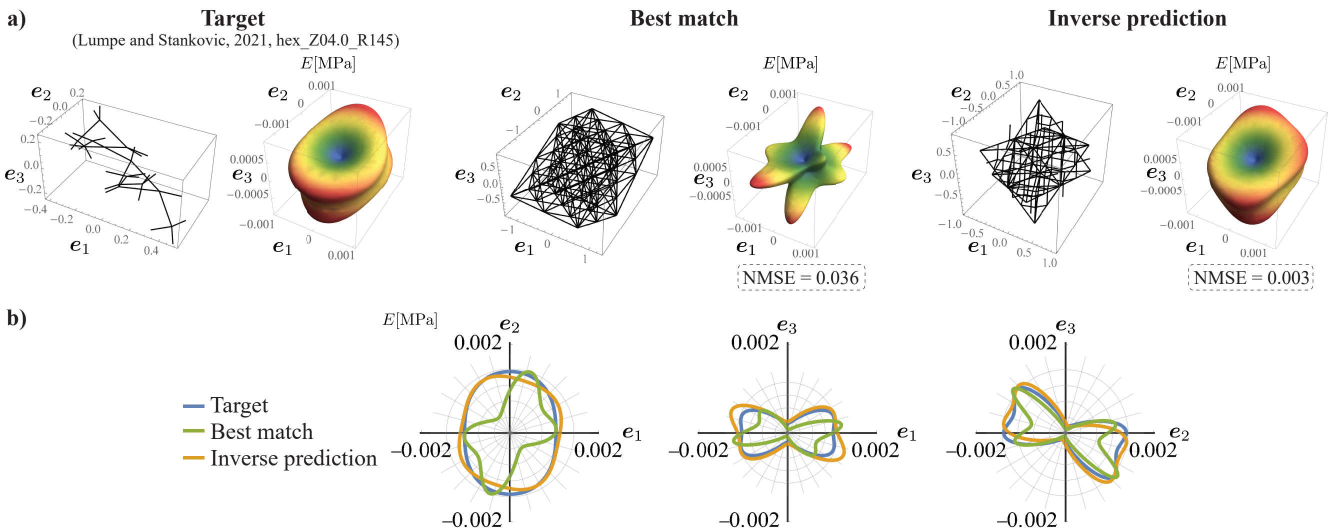

Fig. S7. Comparison of the best match to a given target stiffness in the pre-computed training dataset with the inversely designed lattice. (a) Overview of the topology and elastic surface of the target sample taken from (7), their closest match in terms of minimum mean squared error of the 21 independent elastic parameters, and the inversely designed lattice. **(b)** The superior performance of the inverse design framework is illustrated by projections of the elastic surfaces of the target stiffness, best match in the training dataset, and the inverse prediction onto the $\boldsymbol{e}_1$-$\boldsymbol{e}_2$-, $\boldsymbol{e}_1$-$\boldsymbol{e}_3$-, and $\boldsymbol{e}_2$-$\boldsymbol{e}_3$-planes.

H. Computational efficiency of the inverse model. To estimate the efficiency of our approach, we have added a detailed overview of the computational runtimes required for the training and use of our framework in Table S8. After training is done once *a priori*/offline, subsequent evaluations of the inverse model take a few milliseconds (2ms on average for 30,000 inverse predictions) because no subsequent solution of a complex boundary value problem or an iterative evolution simulation is required—as, e.g., in classical topological optimization and evolutionary algorithms. If one seeks only a single optimal design, then those approaches may indeed be competitive in terms of computational costs. But if one repeatedly needs to evaluate many optimal designs—e.g., when performing multiscale topology optimization (8)—then this makes a dramatic difference. Note that we can easily even reduce the training time if we consider less training data without sacrificing much accuracy (see Table S7) or consider earlier stopping for the inverse model training (since the loss decreases only marginally after around epoch 100, as shown in Figure S6).

Table S8. Runtime, software, and hardware resources used for different tasks (including in parentheses the runtimes for a reduced dataset consisting of 300,000 lattice-stiffness pairs, see also Section 3.F). [2]Reported runtimes are rough estimates only. [3]Tasks were performed on a 2.2 GHz Intel Xeon E5-2650 processor with 256 GB of DDR3 memory at 2.5 GHz. [4]Tasks were performed on a single Nvidia Quadro RTX 6000 with 24 GB GDDR6 memory using CUDA 11. [5]Runtimes for predictions via the NNs are reported for one sample (averaged over a batch size of 1% of the full training data). Note that the averaged runtime partly depends on the size of the evaluated dataset due to the computational overhead.

**Task**	**Software**	**Hardware**	**Runtime**[2]
Generating dataset of lattices and corresponding stiffnesses	In-house C++ FEM code	CPU (10 cores)[3]	15.5 hours (90 minutes)
Training $\mathcal{F}_1$	PyTorch in Python	GPU[4]	4 hours (25 minutes)
Training $\mathcal{F}_2$	PyTorch in Python	GPU[4]	8 hours (50 minutes)
Training $\mathcal{G}_1, \mathcal{G}_2$	PyTorch in Python	GPU[4]	13 hours (80 minutes)
Lattice prediction using $\mathcal{G}_2$[5]	PyTorch in Python	GPU[4]	2 milliseconds

4. Evaluation of the inverse model on crystallographic truss networks and bone samples

The 21 anisotropic stiffness components of the homogenized responses of the truss lattices computed by Lumpe and Stankovic (7) and of the bone samples characterized by Colabella et al. (9), including their inverse-designed counterparts, are presented in Tables S9 and S11, respectively (using Voigt notation for the stiffness components in 3D). As mentioned in the main article, we can leverage the variational nature of our framework to propose a large variety of lattice candidates and subsequently select the ones with the lowest NMSE, which can be evaluated (at negligible computational cost) by the forward model. For this study, we set $\tau = 100$ in the Gumbel-softmax layer to enforce a large exploration of proposed lattices and selected the best predictions from 10,000 generated samples, which only required around one minute of overall runtime.

The inverse model predicts a lattice unit cell based on a normalized stiffness input, i.e., $\mathbb{C} = \mathbb{C}_b/E_b$ for a lattice with the actual homogenized stiffness response $\mathbb{C}_b$ manufactured with an isotropic base material having Young's modulus E_b. Therefore, we normalize the target stiffness by E_b. As reported in the main article, we consider Ti-6Al-4V with $E_b = 114\,\text{GPa}$ (10) for the application in bone implants due to its excellent biocompatibility and corrosion resistance (11), and we hence normalize the reported bone stiffness values accordingly before passing them into the inverse model. Note that, in principle, any E_b can be considered, as long as the given target stiffness can be obtained by lattices within the given limits of relative density ρ.

As the stiffnesses provided by Lumpe and Stankovic (7) were already normalized by the base material's Young's modulus, no further pre-processing was required. Nevertheless, we applied a factor of 20 to the provided stiffness of hex_Z04.0_R145, as the original stiffness is not obtainable within the density range of our design space (as it would require relative densities below our chosen lower bound of $\rho = 0.002$, which can easily be assessed as the corresponding inverse predictions approach the bounds of our design space). Alternatively, one could also retrain the model with an even lower bound on the relative density and subsequently omit the scaling.

To give further quantitative metrics for the performance of our framework, we have applied it to the anisotropic stiffnesses of the complete catalog of crystallographic period networks reported in (7), consisting of 17,262 vastly different truss topologies (with notable shares of highly symmetric (i.e., cubic) to less symmetric (i.e., monoclinic) structures). As before, we have uniformly scaled the stiffnesses by a factor of 10, since some of the provided structures are highly compliant and would otherwise require relative densities below the lower limit of our design space. The results are shown in the histogram in Figure S8. The vast majority of predictions shows an NMSE comparable to the predictions shown in Figure 3 of the main article, highlighting that they do give an accurate representation of the performance of our inverse model on arbitrary stiffnesses.

Table S9. Elastic constants (using Voigt notation) of crystallography-inspired periodic truss networks and the corresponding elastic constants of the lattices obtained by our inverse design framework, visualized in Figure 3a of the main article. The design parameters of the inverse predictions are listed in Table S10.

	Computed by Lumpe and Stankovic (7) [kPa]	Inverse design [kPa]
trig_Z15.5_E1136 Prediction A	$\begin{bmatrix} 2.39 & 0.80 & 0.56 & -0.23 & 0 & 0 \\ 0.80 & 2.39 & 0.56 & 0.23 & 0 & 0 \\ 0.56 & 0.56 & 1.40 & 0 & 0 & 0 \\ -0.23 & 0.23 & 0 & 0.56 & 0 & 0 \\ 0 & 0 & 0 & 0 & 0.56 & -0.23 \\ 0 & 0 & 0 & 0 & -0.23 & 0.80 \end{bmatrix}$	$\begin{bmatrix} 2.24 & 0.81 & 0.57 & -0.25 & 0.07 & 0.12 \\ 0.81 & 2.47 & 0.55 & 0.02 & -0.08 & -0.06 \\ 0.57 & 0.55 & 1.43 & -0.13 & -0.01 & -0.08 \\ -0.25 & 0.02 & -0.13 & 0.55 & -0.08 & -0.08 \\ 0.07 & -0.08 & -0.01 & -0.08 & 0.58 & -0.25 \\ 0.12 & -0.06 & -0.08 & -0.08 & -0.25 & 0.81 \end{bmatrix}$
trig_Z15.5_E1136 Prediction B	$\begin{bmatrix} 2.39 & 0.80 & 0.56 & -0.23 & 0 & 0 \\ 0.80 & 2.39 & 0.56 & 0.23 & 0 & 0 \\ 0.56 & 0.56 & 1.40 & 0 & 0 & 0 \\ -0.23 & 0.23 & 0 & 0.56 & 0 & 0 \\ 0 & 0 & 0 & 0 & 0.56 & -0.23 \\ 0 & 0 & 0 & 0 & -0.23 & 0.80 \end{bmatrix}$	$\begin{bmatrix} 2.42 & 0.75 & 0.59 & -0.25 & 0.01 & 0.07 \\ 0.75 & 2.68 & 0.51 & 0.12 & -0.06 & -0.06 \\ 0.59 & 0.51 & 1.62 & 0.03 & -0.07 & 0.02 \\ -0.25 & 0.12 & 0.03 & 0.51 & 0.02 & -0.06 \\ 0.01 & -0.06 & -0.07 & 0.02 & 0.59 & -0.25 \\ 0.07 & -0.06 & 0.02 & -0.06 & -0.25 & 0.75 \end{bmatrix}$
hex_Z04.0_R145 Prediction A	$\begin{bmatrix} 2.90 & 1.94 & 1.33 & 0.55 & 0 & 0 \\ 1.94 & 2.85 & 1.18 & 0.55 & 0 & 0 \\ 1.33 & 1.18 & 1.09 & 0.57 & 0 & 0 \\ 0.55 & 0.55 & 0.57 & 0.51 & 0 & 0 \\ 0 & 0 & 0 & 0 & 0.31 & 0 \\ 0 & 0 & 0 & 0 & 0 & 0.47 \end{bmatrix}$	$\begin{bmatrix} 2.95 & 1.93 & 1.31 & 0.53 & 0.06 & 0.02 \\ 1.93 & 2.83 & 1.22 & 0.68 & -0.00 & -0.06 \\ 1.31 & 1.22 & 1.20 & 0.61 & -0.02 & -0.03 \\ 0.53 & 0.68 & 0.61 & 0.47 & -0.01 & -0.02 \\ 0.06 & -0.00 & -0.02 & -0.01 & 0.39 & 0.07 \\ 0.02 & -0.06 & -0.03 & -0.02 & 0.07 & 0.46 \end{bmatrix}$
hex_Z04.0_R145 Prediction B	$\begin{bmatrix} 2.90 & 1.94 & 1.33 & 0.55 & 0 & 0 \\ 1.94 & 2.85 & 1.18 & 0.55 & 0 & 0 \\ 1.33 & 1.18 & 1.09 & 0.57 & 0 & 0 \\ 0.55 & 0.55 & 0.57 & 0.51 & 0 & 0 \\ 0 & 0 & 0 & 0 & 0.31 & 0 \\ 0 & 0 & 0 & 0 & 0 & 0.47 \end{bmatrix}$	$\begin{bmatrix} 3.04 & 2.07 & 1.16 & 0.48 & -0.03 & 0.01 \\ 2.07 & 2.78 & 1.20 & 0.55 & -0.02 & 0.02 \\ 1.16 & 1.20 & 0.99 & 0.43 & 0.02 & 0.02 \\ 0.48 & 0.55 & 0.43 & 0.43 & 0.02 & -0.05 \\ -0.03 & -0.02 & 0.02 & 0.02 & 0.32 & 0.09 \\ 0.01 & 0.02 & 0.02 & -0.05 & 0.09 & 0.42 \end{bmatrix}$

Table S10. Overview of the inverse design parameters Θ^{pred} for those lattices mimicking the crystallography-inspired periodic truss networks, whose elastic constants are presented in Table S9. Rotations R_I and R_{II} are given in angle-axis representation, where θ indicates the rotation angle about the normalized rotation axis (e_1, e_2, e_3) with $e_3 = \sqrt{1 - e_1^2 - e_2^2}$.

Design parameters Θ	trig_Z15.5_E1136 Prediction A	trig_Z15.5_E1136 Prediction B	hex_Z04.0_R145 Prediction A	hex_Z04.0_R145 Prediction B
First elementary topology	1	2	2	1
Tesselation of first topology	1	2	1	1
Second elementary topology	2	5	6	4
Tesselation of second topology	2	2	1	1
Third elementary topology	5	6	6	6
Tesselation of third topology	1	1	2	2
Relative density ρ	1.00%	1.04%	1.63%	1.62%
Stretch U_1, U_2, U_3	$\{1.07, 1.07, 0.94\}$	$\{1.08, 1.04, 0.99\}$	$\{1.09, 1.15, 1.05\}$	$\{1.15, 1.14, 0.99\}$
Stretch V_1, V_2, V_3	$\{1.24, 1.33, 0.95\}$	$\{1.23, 1.33, 0.97\}$	$\{1.29, 1.39, 0.85\}$	$\{1.33, 1.38, 0.84\}$
Rotation $\boldsymbol{R}_I$ $\{\theta, e_1, e_2\}$	$\{1.94, -0.94, 0.31\}$	$\{2.02, -0.87, 0.40\}$	$\{1.95, -0.46, 0.73\}$	$\{2.47, -0.60, 0.67\}$
Rotation $\boldsymbol{R}_{II}$ $\{\theta, e_1, e_2\}$	$\{2.88, 0.54, -0.84\}$	$\{-2.98, -0.52, 0.85\}$	$\{2.92, -0.36, 0.90\}$	$\{2.93, -0.57, 0.79\}$

Table S11. Elastic constants (using Voigt notation) of trabecular bone in bovine femurs and the corresponding elastic constants of the lattices obtained by our inverse design framework, visualized in Figure 3a of the main article. The design parameters of the inverse predictions are listed in Table S12.

	Measured by Colabella et al. (9) [GPa]	Inverse design [GPa]
Trab. bone sample #3 Prediction A	$\begin{bmatrix} 0.57 & 0.23 & 0.22 & -0.01 & -0.02 & 0.00 \\ 0.23 & 0.64 & 0.25 & -0.01 & -0.02 & 0.01 \\ 0.22 & 0.25 & 1.26 & -0.02 & 0.02 & -0.00 \\ -0.01 & -0.01 & -0.02 & 0.32 & -0.02 & -0.00 \\ -0.02 & -0.02 & 0.02 & -0.02 & 0.25 & 0.05 \\ 0.00 & 0.01 & -0.00 & -0.00 & 0.05 & 0.22 \end{bmatrix}$	$\begin{bmatrix} 0.62 & 0.21 & 0.22 & 0.01 & 0.00 & -0.01 \\ 0.21 & 0.69 & 0.26 & -0.05 & -0.01 & -0.00 \\ 0.22 & 0.26 & 1.30 & 0.04 & 0.03 & 0.00 \\ 0.01 & -0.05 & 0.04 & 0.26 & 0.00 & -0.01 \\ 0.00 & -0.01 & 0.03 & 0.00 & 0.22 & 0.01 \\ -0.01 & -0.00 & 0.00 & -0.01 & 0.01 & 0.22 \end{bmatrix}$
Trab. bone sample #3 Prediction B	$\begin{bmatrix} 0.57 & 0.23 & 0.22 & -0.01 & -0.02 & 0.00 \\ 0.23 & 0.64 & 0.25 & -0.01 & -0.02 & 0.01 \\ 0.22 & 0.25 & 1.26 & -0.02 & 0.02 & -0.00 \\ -0.01 & -0.01 & -0.02 & 0.32 & -0.02 & -0.00 \\ -0.02 & -0.02 & 0.02 & -0.02 & 0.25 & 0.05 \\ 0.00 & 0.01 & -0.00 & -0.00 & 0.05 & 0.22 \end{bmatrix}$	$\begin{bmatrix} 0.60 & 0.22 & 0.25 & 0.01 & -0.02 & -0.03 \\ 0.22 & 0.61 & 0.26 & 0.02 & -0.00 & 0.00 \\ 0.25 & 0.26 & 1.24 & -0.05 & 0.01 & -0.01 \\ 0.01 & 0.02 & -0.05 & 0.27 & -0.01 & -0.00 \\ -0.02 & -0.00 & 0.01 & -0.01 & 0.25 & 0.01 \\ -0.03 & 0.00 & -0.01 & -0.00 & 0.01 & 0.22 \end{bmatrix}$
Trab. bone sample #4 Prediction A	$\begin{bmatrix} 0.16 & 0.08 & 0.07 & -0.01 & -0.02 & 0.01 \\ 0.08 & 0.35 & 0.13 & -0.02 & -0.01 & 0.02 \\ 0.07 & 0.13 & 0.61 & 0.03 & -0.00 & -0.03 \\ -0.01 & -0.02 & 0.03 & 0.15 & -0.02 & 0.04 \\ -0.02 & -0.01 & -0.00 & -0.02 & 0.09 & -0.01 \\ 0.01 & 0.02 & -0.03 & 0.04 & -0.01 & 0.11 \end{bmatrix}$	$\begin{bmatrix} 0.19 & 0.09 & 0.07 & -0.00 & -0.01 & 0.00 \\ 0.09 & 0.38 & 0.15 & -0.02 & 0.03 & 0.03 \\ 0.07 & 0.15 & 0.56 & 0.03 & 0.01 & -0.03 \\ -0.00 & -0.02 & 0.03 & 0.15 & -0.03 & 0.03 \\ -0.01 & 0.03 & 0.01 & -0.03 & 0.07 & -0.00 \\ 0.00 & 0.03 & -0.03 & 0.03 & -0.00 & 0.09 \end{bmatrix}$
Trab. bone sample #4 Prediction B	$\begin{bmatrix} 0.16 & 0.08 & 0.07 & -0.01 & -0.02 & 0.01 \\ 0.08 & 0.35 & 0.13 & -0.02 & -0.01 & 0.02 \\ 0.07 & 0.13 & 0.61 & 0.03 & -0.00 & -0.03 \\ -0.01 & -0.02 & 0.03 & 0.15 & -0.02 & 0.04 \\ -0.02 & -0.01 & -0.00 & -0.02 & 0.09 & -0.01 \\ 0.01 & 0.02 & -0.03 & 0.04 & -0.01 & 0.11 \end{bmatrix}$	$\begin{bmatrix} 0.19 & 0.10 & 0.07 & -0.01 & -0.03 & -0.01 \\ 0.10 & 0.38 & 0.13 & -0.04 & 0.04 & 0.01 \\ 0.07 & 0.13 & 0.59 & 0.04 & 0.00 & -0.03 \\ -0.01 & -0.04 & 0.04 & 0.13 & -0.03 & 0.04 \\ -0.03 & 0.04 & 0.00 & -0.03 & 0.07 & -0.01 \\ -0.01 & 0.01 & -0.03 & 0.04 & -0.01 & 0.10 \end{bmatrix}$

Table S12. Overview of the inverse designed lattices Θ^{pred} for those lattices mimicking the bovine femoral specimens, whose elastic constants are presented in Table S11. Rotations R_I and R_{II} are given in angle-axis representation, where θ indicates the rotation angle about the normalized rotation axis (e_1, e_2, e_3) with $e_3 = \sqrt{1 - e_1^2 - e_2^2}$.

Design parameters $\boldsymbol{\Theta}$	Trab. bone #3 Prediction A	Trab. bone #3 Prediction B	Trab. bone #4 Prediction A	Trab. bone #4 Prediction B
First elementary topology	1	1	2	2
Tesselation of first topology	2	2	2	2
Second elementary topology	2	2	3	3
Tesselation of second topology	2	1	1	2
Third elementary topology	4	5	5	5
Tesselation of third topology	1	1	2	1
Relative density ρ	3.50%	3.43%	1.54%	1.55%
Stretch U_1, U_2, U_3	$\{1.37, 1.14, 1.09\}$	$\{1.51, 1.06, 0.98\}$	$\{1.20, 1.04, 1.04\}$	$\{1.17, 1.05, 1.05\}$
Stretch V_1, V_2, V_3	$\{1.15, 1.49, 0.97\}$	$\{1.24, 1.42, 1.16\}$	$\{1.24, 1.42, 0.81\}$	$\{1.25, 1.45, 0.81\}$
Rotation $\boldsymbol{R}_I$ $\{\theta, e_1, e_2\}$	$\{1.57, -0.31, 0.72\}$	$\{2.07, -0.29, 0.68\}$	$\{1.91, -0.24, 0.59\}$	$\{1.70, -0.26, 0.63\}$
Rotation $\boldsymbol{R}_{II}$ $\{\theta, e_1, e_2\}$	$\{-2.45, -0.44, 0.70\}$	$\{2.90, -0.31, 0.90\}$	$\{-2.27, -0.63, 0.52\}$	$\{-2.10, -0.65, 0.53\}$

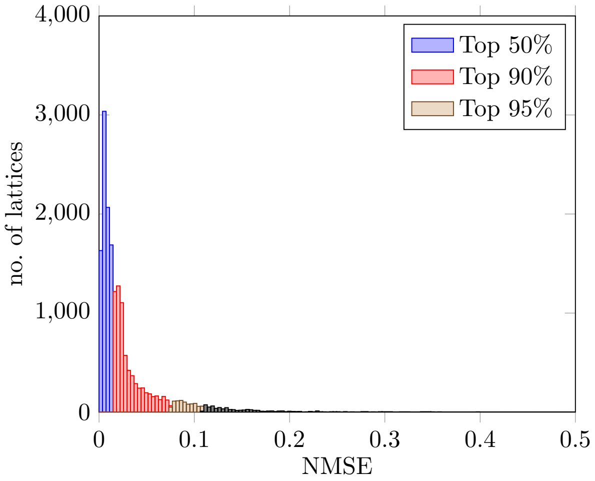

Fig. S8. Histogram of the NMSEs of the inversely generated designs based on the homogenized stiffnesses of the crystallographic period networks (consisting of 17,262 different topologies) provided in (7). Different percentiles are color-coded to better interpret the evaluated NMSEs.

5. Generation of functionally graded lattices

For the integration of different truss UCs in a functionally graded lattice with spatially varying truss topology and geometry, we must, on the one hand, transition between different UC topologies (which here were chosen to all be based on a cubic UC). On the other hand, we must transition between differently shaped UCs arising from the application of affine transformations and rotations. In practice, we choose a total of k control points at locations $\{\boldsymbol{x}_1, \dots, \boldsymbol{x}_k\}$, at which we define a local truss UC (along with its topology and geometry), so that we seek to smoothly transition between those.

All lattices are by definition periodic, and the domain Ω_{UC} of each UC is spanned by three translation vectors forming the basis $\mathcal{A} = \{\boldsymbol{a}_1, \boldsymbol{a}_2, \boldsymbol{a}_3\}$ in 3D, which defines the tessellation of the UC into the truss lattice. Let us denote by $\mathcal{K} = \{\boldsymbol{k}_1, \boldsymbol{k}_2, \boldsymbol{k}_3\}$ the triad of vectors forming the basis of the corresponding reciprocal lattice.

Any periodic (simple Bravais) lattice is represented equally by the so-called impulse train and its Fourier representation:

$$\tau(\boldsymbol{x}) = \prod_{i=1}^{3} \sum_{n=-\infty}^{\infty} \delta\left(\frac{\boldsymbol{k}_i \cdot \boldsymbol{x}}{||\boldsymbol{k}_i||} - n||\boldsymbol{k}_i||\right) = \prod_{i=1}^{3} \frac{1}{1+M}\left[1 + \sum_{m=1}^{M} \cos(2\pi m \boldsymbol{k}_i \cdot \boldsymbol{x})\right], \quad [12]$$

where δ denotes the Dirac delta function, and $M > 0$ is an integer.

In a spatially variant lattice, the above Bravais basis vectors vary from point to point, i.e., we assume a spatially varying basis $\mathcal{A}(\boldsymbol{x}) = \{\boldsymbol{a}_1(\boldsymbol{x}), \boldsymbol{a}_2(\boldsymbol{x}), \boldsymbol{a}_3(\boldsymbol{x})\}$ and consequently a spatially varying reciprocal basis $\mathcal{K}(\boldsymbol{x}) = \{\boldsymbol{k}_1(\boldsymbol{x}), \boldsymbol{k}_2(\boldsymbol{x}), \boldsymbol{k}_3(\boldsymbol{x})\}$. Note that we tacitly assume a separation of scales between the microscale (i.e., the scale of the truss UC dimensions) and the

macroscale (i.e., the scale of the overall body to be filled with the spatially graded truss), which allows us to assign a unique basis $\mathcal{A}$ (and hence $\mathcal{K}$) to each point $\boldsymbol{x}$ in the macroscale body. Of course, in reality such a separation of scales may not be realizable, yet the resulting framework serves as an effective approximation whenever the lattice bases vary sufficiently smoothly across the macroscale body.

To realize a spatially variant truss lattice, we aim to construct a (non-periodic) truss with smoothly varying topology such as to approximate $\mathcal{A}(\boldsymbol{x})$ locally. To this end, we relax the above description of the Fourier-transformed impulse train by introducing three projection functions $\phi_i(\boldsymbol{x})$, one for each of the three $\boldsymbol{k}_i$-vectors in $\mathcal{K}$. Further, we note that the translational symmetry is contained within the first spatial harmonic, which is why we simplify the Fourier decomposition by truncating after its first spatial harmonic ($M = 1$), resulting in the approximation

$$\tau'(\boldsymbol{x}) = \frac{1}{8}\prod_{i=1}^{3}\left[1+\cos\big(2\pi\mu\phi_i(\boldsymbol{x})\big)\right] \qquad \text{with} \qquad \phi_i(\boldsymbol{x}) = \arg\inf \int_\Omega ||\nabla\phi_i(\boldsymbol{x}) - \boldsymbol{k}_i||^2 \,\mathrm{d}\Omega. \tag{13}$$

$\phi_i(\boldsymbol{x})$ is hence a smoothly varying field that is locally tangential to $\boldsymbol{k}_i$, such that the set $\{\phi_1(\boldsymbol{x}), \phi_2(\boldsymbol{x}), \phi_3(\boldsymbol{x})\}$ presents an approximate, smooth, spatially varying basis field over Ω.

Now, consider a desired basis vector field $\mathcal{A}(\boldsymbol{x})$ (which, e.g., locally matches the translation vectors $\{\boldsymbol{a}_1, \boldsymbol{a}_2, \boldsymbol{a}_3\}$ of the truss UCs obtained from the inverse model applied to different properties at different control points $\boldsymbol{x}_i$ on a body Ω). In practice, we define a radial basis function $N(\boldsymbol{x}, \boldsymbol{x}_i)$ for each control point $\boldsymbol{x}_i$, centered at control point $\boldsymbol{x}_i$, as

$$N(\boldsymbol{x}, \boldsymbol{x}_i) = \frac{e^{-\zeta\|\boldsymbol{x}-\boldsymbol{x}_i\|^2}}{\sum_{j=1}^{k} e^{-\zeta\|\boldsymbol{x}-\boldsymbol{x}_j\|^2}}. \tag{14}$$

$\zeta > 0$ controls the thickness of the transition layer between different topologies. If $\mathcal{A}_k$ is the local basis of the UC translation vectors at control point k, then we interpolate to obtain

$$\mathcal{A}(\boldsymbol{x}) = \sum_{i=1}^{k} \mathcal{A}_i\, N(\boldsymbol{x}, \boldsymbol{x}_i). \tag{15}$$

For example, in Figure 3b in the main article, we introduced two control points on the x_1-axis at $\boldsymbol{x}_1 = (x_{min}, 0, 0)^T$ and $\boldsymbol{x}_2 = (x_{max}, 0, 0)^T$, and we chose $\zeta = 0.5$.

Given $\mathcal{A}(\boldsymbol{x})$, we numerically solve the least-squares problem in Eq. (13) for the projection functions $\phi_i(\boldsymbol{x})$, using the finite element method. Subsequently, we construct the relaxed lattice function $\tau'(\boldsymbol{x})$ and choose all N local minima of $\tau'(\boldsymbol{x})$ in the domain Ω as the set of the UC centroid positions, $\mathcal{B}_{\mathrm{UC}}^{\Omega} = \{\boldsymbol{x}_1, \ldots, \boldsymbol{x}_N\}$. Finally, we assemble the global lattice by placing a UC at each $\boldsymbol{x}_\nu \in \mathcal{B}_{\mathrm{UC}}^{\Omega}$ with basis $\mathcal{A}(\boldsymbol{x}_\nu)$. Note that the corner nodes of the resulting adjacent UCs do not perfectly overlap. We therefore apply a smoothing step, which replaces all almost overlapping nodes by a single new node at the arithmetic mean position of the original nodes, and we adjust the beam connectivities accordingly.

While the above strategy takes care of spatially varying translation vectors, we must also transition between the different UC topologies at the k control points. To this end, we superimpose all k UC topologies and weight the diameter $d^s(\boldsymbol{x})$ (where $s = 1, ..., m$) of each of the m individual struts in the superimposed UC by a linear superposition of the constituent diameters by re-using radial basis functions Eq. (14), such that

$$d^s(\boldsymbol{x}) = \sum_{i=1}^{k} d_i^s\, N(\boldsymbol{x}, \boldsymbol{x}_i). \tag{16}$$

Here, d_i^s corresponds to the respective strut diameter in the UC at control point $\boldsymbol{x}_i$ (where $d_i^s = d_i$ if the strut is included in the topology with diameter d_i and $d_i^s = 0$ if it is not). In Figure 3b in the main article, the two control points at $\boldsymbol{x}_1 = (x_{min}, 0, 0)^T$ and $\boldsymbol{x}_2 = (x_{max}, 0, 0)^T$ reduce Eq. (16) to

$$d^s(x_1) = [1 - \lambda(x_1)]\, d_1^s + \lambda(x_1) d_2^s \qquad \text{with} \qquad \lambda(x_1) = \frac{e^{-\zeta[x_1-(x_{max}-x_{min})]^2}}{e^{-\zeta x_1^2} + e^{-\zeta[x_1-(x_{max}-x_{min})]^2}}, \tag{17}$$

and we again chose $\zeta = 0.5$.

References

1. Glaesener RN, Träff EA, Telgen B, Canonica RM, Kochmann DM (2020) Continuum representation of nonlinear three-dimensional periodic truss networks by on-the-fly homogenization. *International Journal of Solids and Structures* 206:101–113.
2. Zhou Y, Barnes C, Lu J, Yang J, Li H (2019) On the continuity of rotation representations in neural networks. *Proceedings of the IEEE Computer Society Conference on Computer Vision and Pattern Recognition* 2019-June:5738–5746.
3. Bond WL (1943) The Mathematics of the Physical Properties of Crystals. *Bell System Technical Journal* 22(1):1–72.
4. Paszke A, et al. (2019) PyTorch: An imperative style, high-performance deep learning library.

5. Kingma DP, Ba J (2014) Adam: A Method for Stochastic Optimization.
6. Wes McKinney (2010) Data Structures for Statistical Computing in Python in *Proceedings of the 9th Python in Science Conference*, eds. Stéfan van der Walt, Jarrod Millman. pp. 56 – 61.
7. Lumpe TS, Stankovic T (2021) Exploring the property space of periodic cellular structures based on crystal networks. *Proceedings of the National Academy of Sciences* 118(7):e2003504118.
8. Zheng L, Kumar S, Kochmann DM (2021) Data-driven topology optimization of spinodoid metamaterials with seamlessly tunable anisotropy. *Computer Methods in Applied Mechanics and Engineering* 383:113894.
9. Colabella L, Cisilino AP, Häiat G, Kowalczyk P (2017) Mimetization of the elastic properties of cancellous bone via a parameterized cellular material. *Biomechanics and Modeling in Mechanobiology* 16(5):1485–1502.
10. Bandyopadhyay A, et al. (2010) Influence of porosity on mechanical properties and in vivo response of Ti6Al4V implants. *Acta Biomaterialia* 6(4):1640–1648.
11. Tan XP, Tan YJ, Chow CS, Tor SB, Yeong WY (2017) Metallic powder-bed based 3D printing of cellular scaffolds for orthopaedic implants: A state-of-the-art review on manufacturing, topological design, mechanical properties and biocompatibility. *Materials Science and Engineering C* 76:1328–1343.
